# Prophylactic Aspirin Dose and Preeclampsia

**DOI:** 10.1001/jamanetworkopen.2024.57828

**Published:** 2025-02-03

**Authors:** Ellen Kupka, Susanne Hesselman, Jóhanna Gunnarsdóttir, Anna-Karin Wikström, Catherine Cluver, Stephen Tong, Roxanne Hastie, Lina Bergman

**Affiliations:** 1 Department of Obstetrics and Gynecology, Institute of Clinical Science, Sahlgrenska Academy, University of Gothenburg, Gothenburg, Sweden; 2 Department of Research and Higher Education, Center for Clinical Research, Dalarna, Uppsala University, Region Dalarna, Falun, Sweden; 3 Department of Women’s and Children’s health, Uppsala University, Uppsala, Sweden; 4 Faculty of Medicine, University of Iceland, Reykjavík, Iceland; 5 Department of Obstetrics and Gynecology, Landspítali The National University Hospital of Iceland, Reykjavík, Iceland; 6 Mercy Perinatal, Mercy Hospital for Women, Melbourne, Victoria, Australia; 7 Department of Obstetrics and Gynaecology, Stellenbosch University, Cape Town, South Africa; 8 Department of Obstetrics and Gynaecology, University of Melbourne, Heidelberg, Victoria, Australia

## Abstract

**Importance:**

It is unclear whether a higher dose (150-160 mg) or a lower dose (75 mg) of aspirin should be used to prevent preeclampsia.

**Objectives:**

To compare the risk of preeclampsia and bleeding complications between women using 150 to 160 mg of aspirin and those using 75 mg of aspirin for preeclampsia prevention.

**Design, Setting, and Participants:**

This nationwide cohort study included 13 828 women giving birth at 22 weeks’ gestation or later in Sweden between January 2017 and December 2020 who used low dose aspirin (75-160 mg) during pregnancy. Data were analyzed from October to November 2023.

**Exposure:**

The use of 150 to 160 mg or 75 mg of aspirin in pregnancy.

**Main Outcome and Measures:**

The main outcome was a preeclampsia diagnosis recorded in the maternal birth record at the time of hospital discharge. The main safety outcome was postpartum hemorrhage, defined as bleeding more than 1000 mL after delivery. Relative risks (RRs) and 95% CIs were estimated using a doubly robust inverse probability–weighted regression adjustment model controlling for background characteristics.

**Results:**

In the total cohort of 13 828 women, the mean (SD) age was 33.0 (5.5) years and 3003 women (21.7%) were nulliparous. Of the women, 4687 (33.9%) were prescribed 150 to 160 mg of aspirin, and 9141 (66.1%) were prescribed 75 mg of aspirin. A total of 10 635 women (76.9%) had at least 2 dispensed prescriptions of low-dose aspirin. Among women using 150 to 160 mg of aspirin, 443 (9.5%) developed preeclampsia compared with 812 (8.9%) of those using 75 mg of aspirin (adjusted RR [aRR], 1.07; 95% CI, 0.93-1.24). Additionally, the risk of postpartum hemorrhage between the groups was similar, with 326 women (6.9%) using 150 to 160 mg of aspirin experiencing a postpartum hemorrhage compared with 581 (6.4%) in the 75-mg group (aRR, 1.08; 95% CI, 0.90-1.30).

**Conclusions and Relevance:**

In this cohort study of 13 828 women, no difference was found in preeclampsia incidence or bleeding complications between those using 150 to 160 mg of aspirin vs 75 mg of aspirin during pregnancy for preeclampsia prevention. These findings suggest that either dose may be a reasonable choice when using aspirin to prevent preeclampsia. However, large randomized trials investigating aspirin dose in pregnancy are still needed.

## Key Points

**Question:**

Is higher dose aspirin (150-160 mg) associated with an altered risk of preeclampsia or bleeding complications compared with lower dose aspirin (75 mg)?

**Findings:**

This cohort study of 13 828 pregnant women found no association between aspirin dose (150-160 mg vs 75 mg) and the risk of developing preeclampsia or bleeding complications.

**Meaning:**

These findings suggest no difference in the risk of preeclampsia or bleeding complications by aspirin dose.

## Teaser

This cohort study evaluates the risk of preeclampsia and bleeding complications between different aspirin dosages for preeclampsia prevention.

## Introduction

Preeclampsia is a severe complication of pregnancy defined as the new onset or worsening of hypertension after 20 weeks’ gestation combined with proteinuria or maternal organ dysfunction.^1^ It affects about 5% of pregnancies worldwide.^2,3^ After postpartum hemorrhage, preeclampsia is the leading direct cause of maternal mortality.^3^

Low-dose aspirin is the only drug with high level evidence for preventing preeclampsia. Still, there is no consensus on the optimal dose of aspirin to prevent preeclampsia with limited evidence available to guide this decision. Consequently, international guidelines differ greatly. Some suggest less than 100 mg (mainly 75-81 mg) is sufficient, while others recommend a dose higher than 100 mg (often at 150-162 mg). Some make no suggestion of a specific dose beyond recommending low-dose aspirin.^4,5,6^

Higher doses (150-160 mg) are often recommended with the hypothesis of being more effective at preventing preeclampsia compared with lower doses. However, there are no large randomized clinical trials directly comparing doses and meta-analyses pooling other study types have been inconclusive.^7^

There is also evidence that aspirin may increase the risk of bleeding. Using Swedish population-based data, we have previously reported aspirin use in pregnancy to be associated with an increased likelihood of postpartum hemorrhage, defined as blood loss greater than 1000 mL (odds ratio, 1.23; 95% CI, 1.08-1.39).^8^ Since this report, several other studies, including a meta-analysis, have confirmed aspirin is associated with an increased risk of bleeding during pregnancy.^9^ There are no adequately powered studies directly comparing bleeding risk between higher and lower doses of aspirin.

Sweden offers a unique opportunity to investigate the outcomes of different aspirin doses in pregnancy at a population level. The Swedish national registers offer reliable data both regarding the exposure (aspirin in different dosages) and the outcome (preeclampsia). In addition, there are national Swedish guidelines for aspirin treatment during pregnancy. Lastly, both 75-mg and 150- to 160-mg doses of aspirin have been prescribed to women in Sweden at increased risk of preeclampsia. We therefore used national level Swedish data to compare 150 to 160 mg with 75 mg of aspirin during pregnancy, and to evaluate the association with preeclampsia and bleeding complications.

## Methods

### Design and Participants

The study was approved by the ethical review board at Uppsala University. According to current Swedish regulation, no informed consent is required for research using national registry data. This was a Swedish register–based cohort study performed in accordance with the Strengthening the Reporting of Observational Studies in Epidemiology (STROBE) reporting guidelines.^10^ Further information on the methods is available in the eMethods in Supplement 1.

Data were collected from the Swedish Medical Birth Register, the Swedish Prescribed Drug Register, the National Patient Register, and the Education Register held by Statistics Sweden. The registers were linked with the personal identity number assigned to each Swedish resident at birth or immigration to Sweden.^11^ The Swedish Medical Birth Register covers more than 98% of all births in Sweden and provides validated data about pregnancies, labor, and perinatal outcomes from diagnoses and standardized medical records.^12^ The Swedish Prescribed Drug Register uses the World Health Organization’s Anatomical Therapeutic Chemical Classification codes and provides patient-level data on all dispensed prescribed drugs in Sweden.^13^ The National Patient Register holds geographic and medical diagnoses for inpatients and outpatients in specialized care.^14^ The education register from Statistics Sweden contains data on the highest level of education obtained.^15^

Women were included if they had a singleton pregnancy and at least 1 dispensed aspirin prescription from 3 months before conception until delivery. Aspirin was seldom prescribed at the dose of 150 to 160 mg before 2017.^16^ Thus, to minimize variations in baseline characteristics and clinical practice, only pregnancies from 2017 to 2020 were included in this study. If a woman had 2 or more pregnancies during this period, 1 pregnancy was randomly selected. To ensure that women were prescribed aspirin for preeclampsia prevention, women with an *International Statistical Classification of Diseases and Related Health Problems, Tenth Revision (ICD-10)* code for previous or current cardiovascular disease (eTable 1 in Supplement 1) were excluded (65 participants).

### Covariates

Covariates included year, maternal characteristics, geographical region of the maternity unit, conception via in vitro fertilization, pregestational disorders, pregnancy complications, mode of delivery, gestational age at delivery, birth weight, small for gestational age infant,^17^ stillbirth, antidepressant, and low-molecular-weight-heparin (LMWH) use. Country of birth, which was included as a factor associated with risk for preeclampsia, is sourced from Statistics Sweden and recorded by administrative personnel for each Swedish citizen at birth or upon immigration. A detailed description of the covariates is provided in the eMethods and eTable 1 in Supplement 1.

### Exposure

The exposure was the use of 150 mg (women with dispensed prescriptions of two 75 mg aspirin doses once a day) or 160 mg of aspirin during pregnancy. The reference group was women using 75 mg of aspirin. Data on aspirin dose were obtained from the first dispensed prescription in the pregnancy, as recorded in the Swedish Prescribed Drug Register (eTable 1 in Supplement 1).

### Outcomes

We included 2 primary outcomes to reflect effectiveness and safety, both determined a priori. For effectiveness, our primary outcome was a diagnosis of preeclampsia recorded in the maternal birth record at the time of hospital discharge. For safety, our main outcome was postpartum hemorrhage, defined as bleeding of more than 1000 ml within 24 hours of delivery.

Secondary outcomes included gestational age at delivery with preeclampsia diagnosis (<37 weeks’ gestation, <34 weeks’ gestation, or ≥37 weeks’ gestation), preeclampsia with a small for gestational age infant (defined in Sweden as birth weight <2.5 percentile^17^), antepartum hemorrhage, intrapartum hemorrhage, postpartum hematoma, neonatal intracranial bleeding, and anemia at time of birth. The information on outcomes was obtained from the Medical Birth Register. For anemia, information was obtained from both the Medical Birth Register and the National Patient Register (eTable 1 in Supplement 1).

### Subgroup Analyses

We conducted stratified analyses by vaginal birth or cesarean delivery for the outcomes of postpartum hemorrhage, intrapartum hemorrhage, postpartum hematoma, neonatal intracranial bleeding, and anemia. We also performed sensitivity analyses for the primary outcomes of preeclampsia and postpartum hemorrhage in nulliparous women (women pregnant with their first child).

### Statistical Analysis

An a priori statistical analysis plan was developed and agreed upon by all authors and is provided in the eAppendix in Supplement 1. Population characteristics were described by aspirin dose (75 mg or 150-160 mg) and summarized using mean (SD), median (IQR), and number (%) according to type and distribution of data.

To investigate the association between aspirin dose (150-160 mg compared with 75 mg [reference group]) and outcomes, a doubly robust inverse probability–weighted regression adjustment (IPWRA) was employed using the teffects suite in StataMP version 18 (StataCorp). This approach minimizes the differences between groups in observational research to better isolate the treatment effect.

Briefly, inverse probability weighting estimates selection to treatment (the treatment model—high or low dose aspirin), then estimates treatment for all observations and assigns the inverse of probability of treatment for treated and inverse-probability of not being treated for controls. The outcome model is then reestimated using these new weights. This is combined with a regression-adjusted outcome model that also includes potential confounding variables. From this, the potential outcome means are obtained for each group and log risk ratios and corresponding 95% CIs determined. Importantly, the IPWRA estimator accounts for misspecification in either the outcome or treatment model and generates a robust estimate. For each outcome, included covariates were determined a priori by the research team and with the use of directed acyclic graphs (eAppendix in Supplement 1).

The covariates in the treatment model were, as far as possible, guided by risk factors outlined in the Swedish National Guideline for preeclampsia prevention^18^ and the Fetal Medicine Foundation risk assessment algorithm.^19^ A logit treatment model was used and included the following covariates: maternal age, body mass index, smoking status, interpregnancy interval, conception via assisted reproduction, country of birth, parity, pregestational disorders, prior birth of a small for gestational age infant, prior placental abruption, prior gestational hypertension with delivery less than 34 weeks, prior stillbirth, prior preeclampsia, year of birth, and region.

Regression adjustment (outcome) models were individually considered for each outcome of interest. For preeclampsia-related outcomes, the regression adjustment included maternal age, body mass index, smoking status, interpregnancy interval, conception via assisted reproduction, country of birth, parity, pregestational disorders, prior pregnancy complications, and prior thromboembolism or use of LMWH. For bleeding-related outcomes, the included covariates were maternal age, body mass index, smoking status, country of birth, pregestational disorders, prior pregnancy complications, prior cesarean birth, prior thromboembolism or use of LMWH, use of antidepressants, placental abruption, placenta previa, placenta accreta, labor dystocia, preeclampsia, mode of birth (not included for antepartum hemorrhage), and neonatal birth weight (not included for antepartum hemorrhage).

After adjusting, each covariate was assessed for balance between exposure groups, with a standardized mean difference less than 0.1 deemed adequate and balance plots visualized (eTable 2 and eFigures 1 and 2 in Supplement 1). This process was repeated for each outcome and subgroup analysis. For subgroup analyses by mode of birth and parity, mode of birth and parity were removed from adjusted models, respectively. Women with missing data for any covariate were excluded from the adjusted analyses. Unadjusted and adjusted results were presented as relative risk (RR) with corresponding 95% CIs.

Stata version 18 (StataCorp) and SPSS Statistics for Windows version 29.0 (IBM Corp) were used for all statistical calculations. Data were analyzed from October to November 2023.

## Results

Our cohort included 13 828 women with a recorded aspirin prescription during pregnancy. The mean (SD) age was 33.0 (5.5) years, and 3003 women (21.7%) were nulliparous. Of these, 4687 women (33.9%) used 150 to 160 mg of aspirin, and 9141 women (66.1%) used 75 mg of aspirin. A flowchart for the study population is presented in the Figure. A total of 10 635 women (76.9%) had at least 2 dispensed prescriptions of low-dose aspirin. The number of women with at least 2 dispensed prescriptions was 3781 among women using 150 to 160 mg of aspirin (80.7%) and 6905 among women using 75 mg (75.5%).

**Figure.**
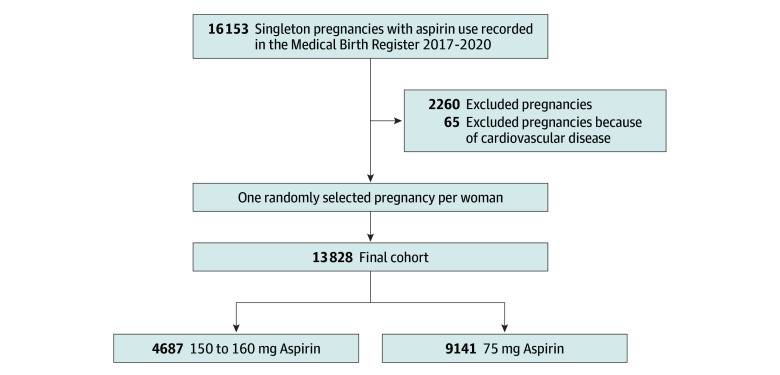
Study Population Flowchart

Compared with those using 75 mg of aspirin, those taking 150 to 160 mg of aspirin were more likely to have a body mass index (calculated as weight in kilograms divided by height in meters squared) above 30 (1422 patients [32%] vs 1990 patients [23%]), be nulliparous (1422 patients [30%] vs 1583 patients [17%]), and to have a vaginal birth (3477 patients [74%] vs 6097 patients [67%]) (Table 1). They were also more likely to have experienced preeclampsia in a previous pregnancy (1327 patients [41%] vs 2544 patients [34%]) (eTable 3 in Supplement 1). Prescription of 150 to 160 mg and 75 mg of aspirin differed by region in Sweden and by year of birth (eTables 4 and 5 in Supplement 1).

**Table 1.** Maternal Characteristics by Aspirin Dosage

Characteristic	Patients, No. (%)		
	Total births (N = 13 828)	Aspirin use	
		150-160 mg (n = 4687)	75 mg (n = 9141)
Age, mean (SD), y	33.0 (5.5)	32.2 (5.5)	33.5 (5.4)
≥35	5300 (38.3)	1513 (32.3)	3787 (41.4)
Body mass index, median (IQR)^a^	25.6 (7.6)	26.2 (9.0)	25.3 (7.0)
Body mass index ≥30	3412 (25.9)	1422 (31.6)	1990 (23.0)
Parity^b^			
Nulliparous	3003 (21.7)	1420 (30.3)	1583 (17.3)
1-3	9647 (69.8)	2937 (62.7)	6710 (73.4)
≥4	1178 (8.5)	330 (7.0)	848 (9.3)
Country of birth			
Nordic	10 159 (73.5)	3567 (76.1)	6592 (72.1)
Non-Nordic European	799 (5.8)	294 (6.3)	505 (5.5)
Rest of the world^c^	2869 (20.7)	825 (17.6)	2044 (22.4)
Missing	1 (<0.1)	1 (<0.1)	0
Smoking first antenatal visit	533 (4.1)	214 (4.8)	319 (3.7)
Missing	726 (5.3)	256 (5.5)	470 (5.1)
Education			
University	7901 (57.1)	2590 (55.3)	5311 (58.1)
Upper secondary school	3388 (24.5)	1263 (26.9)	2125 (23.2)
<12 y Of school attendance	2480 (17.9)	827 (17.6)	1653 (18.1)
Missing	59 (0.4)	7 (0.1)	52 (0.6)
Conception method			
Spontaneous	12 621 (91.3)	4393 (93.7)	8228 (90.0)
Ovulation drugs	227 (1.6)	51 (1.1)	176 (1.9)
In vitro fertilization	1034 (7.5)	254 (5.4)	780 (8.5)
Pregestational disorders			
Chronic hypertension	601 (4.3)	189 (4.0)	412 (4.5)
Type 1 diabetes	488 (3.5)	240 (5.1)	248 (2.7)
Type 2 diabetes	198 (1.4)	92 (2.0)	106 (1.2)
Chronic kidney disease	217 (1.6)	68 (1.5)	149 (1.6)
Systemic lupus erythematosus	206 (1.5)	47 (1.0)	159 (1.7)
Antiphospholipid syndrome	91 (0.7)	7 (0.1)	84 (0.9)
Pregnancy-induced disorders			
Gestational diabetes	881 (6.4)	304 (6.5)	577 (6.3)
Placental abruption	176 (1.3)	54 (1.2)	122 (1.3)
Placenta previa	100 (0.7)	29 (0.6)	71 (0.8)
Gestational age at delivery, median (IQR), wk	39.0 (2.0)	39.0 (2.0)	39.0 (2.0)
Induction of labor	4508 (32.6)	1604 (34.2)	2904 (31.8)
Mode of birth			
Unassisted vaginal	9024 (65.3)	3282 (70.0)	5742 (62.8)
Instrumental vaginal	550 (4.0)	195 (4.2)	355 (3.9)
Cesarean delivery	4254 (30.8)	1210 (25.8)	3044 (33.3)
Use of antidepressants	1176 (8.5)	413 (8.8)	763 (8.3)
Use of low-molecular-weight-heparin^d^	1667 (12.1)	291 (6.2)	1376 (15.1)

^a^ Calculated as weight in kilograms divided by height in meters squared.

^b^ In 2020, our access to registry data was limited to information on parous women.

^c^ Including Africa, Asia, North America, Oceania, the former Soviet Union, South America, and other unspecified countries.

^d^ The use of low-molecular-weight heparin during pregnancy or a thromboembolism diagnosis before the index pregnancy or during the index pregnancy.

### Aspirin Use and Preeclampsia

Among women using 150 to 160 mg of aspirin, 443 (9.5%) developed preeclampsia, compared with 812 of women (8.9%) using 75 mg. This resulted in a nonsignificant crude RR of 1.06 (95% CI, 0.95-1.19) and a similar adjusted RR (aRR) of 1.07 (95% CI, 0.93-1.24) (Table 2).

**Table 2.** Preeclampsia by Aspirin Dosage^a^

Outcome	Patients, No. (%)		Relative risk (95% CI)	
	150-160 mg Aspirin (n = 4687)	75 mg Aspirin (n = 9141)	Crude	Adjusted
Preeclampsia	443 (9.5)	812 (8.9)	1.06 (0.95-1.19)	1.07 (0.93-1.24)
Secondary outcomes				
Preeclampsia with delivery ≥37 wk	313 (6.7)	558 (6.1)	1.09 (0.96-1.25)	0.96 (0.81-1.13)
Preeclampsia with delivery <37 wk	130 (2.8)	254 (2.8)	1.00 (0.81-1.23)	1.31 (0.87-1.95)
Preeclampsia with delivery <34 wk	38 (0.8)	98 (1.1)	0.76 (0.52-1.10)	1.28 (0.77-2.13)
Preeclampsia with small for gestational age infant (≤2.5 percentile)	53 (1.13)	98 (1.1)	1.05 (0.75-1.47)	1.45 (0.93-2.25)

^a^ A total of 13 828 and 12 102 participants included in the crude and adjusted models, respectively. A doubly robust inverse probability–weighted regression adjustment model was used for the adjusted analysis with aspirin 75 mg as reference.

Investigating preeclampsia by timing of birth, we found no difference in the rates of preeclampsia at any gestation. Among women using 150 to 160 mg of aspirin, 313 (6.7%) developed full term preeclampsia, compared with 558 women (6.1%) using 75 mg (aRR, 0.96; 95% CI, 0.81-1.13). Among women using 150 to 160 mg of aspirin, 130 (2.8%) developed preeclampsia with preterm birth at less than 37 weeks’ gestation compared with 254 women (2.8%) using 75 mg (aRR, 1.31; 95% CI, 0.87-1.95). Among women using 150 to 160 mg of aspirin, 38 (0.8%) developed preeclampsia with preterm birth at less than 34 weeks’ gestation, compared with 98 women (1.1%) using 75 mg (aRR, 1.28; 95% CI, 0.77-2.13). Lastly, there was no difference in the risk of developing preeclampsia with small for gestational age infants (53 patients [1.1%] vs 98 patients [1.1%]; aRR, 1.45; 95% CI, 0.93-2.25) (Table 2).

### Aspirin Use and Bleeding Complications

Among women using 150 to 160 mg of aspirin, 326 (6.9%) experienced a postpartum hemorrhage, compared with 581 (6.4%) in the 75-mg group (RR, 1.09; 95% CI, 0.96-1.25). After adjustment, the results remained similar (aRR, 1.08; 95% CI, 0.90-1.30) (Table 3).

**Table 3.** Bleeding Complications by Aspirin Dosage^a^

Outcome	Patients, No. (%)		Relative risk (95% CI)	
	150-160 mg (n = 4687)	75 mg (n = 9141)	Crude	Adjusted
Postpartum hemorrhage	326 (6.9)	581 (6.4)	1.09 (0.96-1.25)	1.08 (0.90-1.30)
Secondary outcomes				
Antepartum hemorrhage	26 (0.6)	49 (0.5)	1.03 (0.64-1.66)	1.36 (0.77-2.40)
Intrapartum hemorrhage	109 (2.3)	293 (3.2)	0.73 (0.58-0.90)	0.96 (0.72-1.30)
Postpartum hematoma	9 (0.2)	27 (0.3)	0.65 (0.31-1.38)	1.82 (0.68-4.89)
Neonatal intracranial bleeding	14 (0.3)	25 (0.3)	1.09 (0.57-2.10)	1.09 (0.49-2.46)
Anemia	558 (11.9)	786 (8.6)	1.38 (1.25-1.53)	1.42 (1.24-1.62)

^a^ A total of 13 828 participants included in the crude model, 12 333 participants included in the adjusted model for postpartum and intrapartum hemorrhage, 12 343 participants included in the adjusted model for antepartum hemorrhage, 12 489 participants included in the adjusted model for hematoma and anemia, and 12 488 participants included in the adjusted model for neonatal intracranial bleeding. A doubly robust inverse probability–weighted regression adjustment model was used for the adjusted analysis with aspirin 75 mg as reference.

Additionally, 150 to 160 mg of aspirin was not associated with altered risk of hemorrhage compared with an aspirin dose of 75 mg. Among women using 150 to 160 mg of aspirin, 26 (0.6%) experienced an antepartum hemorrhage, compared with 49 (0.5%) in the 75-mg group (aRR, 1.36; 95%, CI 0.77-2.40). Among women using 150 to 160 mg of aspirin, 109 (2.3%) experienced an intrapartum hemorrhage, compared with 293 (3.2%) in the 75-mg group (aRR, 0.96; 95% CI, 0.72-1.30). Among women using 150 to 160 mg of aspirin, 9 (0.2%) experienced a postpartum hematoma, compared with 27 (0.3%) in the 75-mg group (aRR, 1.82; 95% CI, 0.68-4.89). Among women using 150 to 160 mg of aspirin, the rate of neonatal intracranial bleeding was 0.3% (14 patients) compared with 0.3% (25 patients) in the 75-mg group (aRR, 1.09; 95% CI, 0.49-2.46). Higher doses of aspirin were associated with a 42% increased risk of anemia diagnosis at birth, with a rate of 558 among women using 150 to 160 mg of aspirin (11.9%) and 786 among women using 75 mg of aspirin (8.6%; aRR, 1.42; 95% CI, 1.24-1.62) (Table 3).

### Subgroup Analyses

#### Vaginal Birth

Among the 9574 women who had a vaginal birth, we found no difference in the risk of postpartum hemorrhage between 150 to 160 mg and 75 mg of aspirin (301 patients [8.7%] vs 484 patients [7.9%]; aRR, 1.51; 95% CI, 0.95-1.40). Results were similar for all bleeding outcomes with no difference between groups. The use of 150 to 160 mg was associated with an increased risk for anemia at birth compared with 75 mg (333 patients [9.6%] vs 414 patients [6.8%]; aRR, 1.32; 95% CI, 1.10-1.56) (Table 4).

**Table 4.** Bleeding Complications by Mode of Birth^a^

Complication	Vaginal birth (n = 9574)				Cesarean birth (n = 4254)			
	Aspirin 150-160 mg (n = 3477), No. (%)	Aspirin 75 mg (n = 6097), No. (%)	Relative risk (95% CI)		Aspirin 150-160 mg (n = 1210), No. (%)	Aspirin 75 mg (n = 3044), No. (%)	Relative risk (95% CI)	
			Crude	Adjusted			Crude	Adjusted
Postpartum hemorrhage	301 (8.7)	484 (7.9)	1.09 (0.95-1.25)	1.51 (0.95-1.40)	25 (2.1)	97 (3.2)	0.65 (0.42-1.01)	0.54 (0.33-0.88)
Intrapartum hemorrhage	5 (0.1)	18 (0.3)	0.49 (0.18-1.31)	0.38 (0.13-1.11)	104 (8.6)	275 (9.0)	0.95 (0.77-1.18)	1.02 (0.76-1.36)
Postpartum hematoma	7 (0.2)	16 (0.3)	0.77 (0.32-1.86)	2.03 (0.62-6.64)	2 (0.2)	11 (0.4)	0.46 (0.10-2.06)	1.20 (0.20-7.25)
Neonatal intracranial bleeding	3 (0.09)	11 (0.2)	0.49 (0.13-1.71)	0.37 (0.09-1.50)	11 (0.9)	14 (0.5)	1.98 (0.90-4.34)	1.78 (0.61-5.18)
Anemia	333 (9.6)	414 (6.8)	1.41 (1.23-1.62)	1.32 (1.10-1.56)	225 (18.6)	372 (12.1)	1.52 (1.31-1.77)	1.51 (1.24-1.84)

^a^ Vaginal birth: 9574 participants included in the crude model; 8580 participants included in the adjusted model for postpartum and intrapartum hemorrhage; and 8720 participants included in the adjusted model for hematoma, neonatal intracranial bleeding, and anemia. Cesarean birth: 4280 participants included in the crude model, 3753 participants included in the adjusted model for postpartum and intrapartum hemorrhage, 3769 participants included in the adjusted model for hematoma and anemia, and 3768 participants included in the adjusted model for neonatal intracranial bleeding. A doubly robust inverse probability weighted regression adjustment model was used for the adjusted analysis with aspirin 75 mg as reference.

#### Cesarean Births

Among the 4254 women who gave birth via cesarean delivery, 150 to 160 mg of aspirin was associated with a reduced risk of postpartum hemorrhage (25 patients [2.1%] vs 97 patients [3.2%]; aRR, 0.54; 95% CI, 0.33-0.88). There was no difference between groups in intrapartum hemorrhage, postpartum hematoma, or neonatal intracranial bleeding. However, the use of 150 to 160 mg of aspirin was associated with an increased risk for anemia at birth when compared with 75 mg of aspirin (225 patients [18.6%] vs 372 patients [12.1%]; aRR, 1.51; 95% CI, 1.24-1.84) (Table 4).

#### Nulliparous Women

Lastly, in a sensitivity analysis restricted to the 3003 nulliparous women, 150 to 160 mg of aspirin was associated with an increased risk of preeclampsia when compared with 75 mg of aspirin (156 patients [11.0%] vs 159 patients [10.0%]; aRR, 1.40; 95% CI, 1.05-1.87) (eTable 6 in Supplement 1). There was no difference in postpartum hemorrhage between the groups (123 patients [8.7%] vs 148 patients [9.4%]; aRR, 1.18; 95% CI, 0.83-1.68) (eTable 6 in Supplement 1).

## Discussion

### Principal Findings

In this cohort study of over 13 000 pregnant women using aspirin, 150 to 160 mg compared with 75 mg of aspirin was not associated with an altered risk of preeclampsia or preterm preeclampsia. Nor was the risk of postpartum hemorrhage changed. Our findings suggest that either dose may be a reasonable choice when using aspirin to prevent preeclampsia.

### Results in the Context of What Is Known

There is limited evidence directly comparing aspirin doses to prevent preeclampsia. As such, the optimal dose is unclear. A 2019 Cochrane review^20^ of antiplatelet agents for the prevention of preeclampsia indirectly compared trials using different doses. They showed a relative risk of preeclampsia of 0.92 (95%, CI, 0.85 to 1.00; 22 618 women; 11 trials) for women using less than 75 mg and 0.78 (95% CI, 0.66 to 0.92; 9107 women; 16 trials) for women using higher doses. A recent meta-analysis^21^ of studies directly comparing aspirin doses including 472 women showed greater benefit for higher doses compared with lower for the prevention of preterm preeclampsia. However, there were significant limitations within these studies with 2 of the 3 included trials not registered and 1 having potential methodological flaws, making the interpretation of these results difficult.^22^

### Clinical Implications

An increasing number of international guidelines recommend aspirin at a dosage of 100 mg or above for preeclampsia prevention. Our study does not provide evidence supporting the superiority of higher doses over lower doses. Concerns have been raised that higher aspirin doses could increase the risk of postpartum hemorrhage and other bleeding complications. However, our findings do not show a clinically important difference in the risk of postpartum hemorrhage between 150 to 160 mg and 75 mg of aspirin.

### Research Implications

Ideally, large, randomized trials comparing different doses should be done to validate our conclusions. Any large trials examining different doses should include both efficacy and safety as primary outcomes to ensure that the risk-benefit ratio can be examined. Trials should include low- and middle-income countries, where the burden of preeclampsia and postpartum hemorrhage is greatest. The Bill and Melinda Gates Foundation recently funded a large trial^23^ in low and middle income country settings comparing 75 and 150 mg of aspirin, including both preeclampsia and hemorrhage outcomes. In addition, a US-based study^24^ was recently funded and plans to investigate the effects of different doses in a population of 10 000 women at increased risk of preeclampsia. Together with our study that included almost 14 000 women, the results from these trials will hopefully provide robust evidence on the appropriate aspirin dosage for preventing preeclampsia.

### Strengths and Limitations

This study has several strengths. It is a large population-based study that leverages data from national registers covering over 98% of the Swedish population that provide reliable data.^12,13^ The *ICD-10* coding for postpartum hemorrhage in Swedish registers is very reliable, with a reported sensitivity of 88.5% and specificity greater than 99%.^25^ Notably, Sweden is 1 of few countries where low-dose aspirin is only available via prescription and not over the counter during pregnancy, ensuring we were able to accurately obtain exposure data. Our statistical method was optimized and provided a doubly robust model, ensuring consistent results even if 1 model (treatment or outcome) was misspecified.

There are some limitations to our study. There were differences in baseline clinical demographics between the groups we compared. Using an IPWRA model, we adjusted for variables associated with treatment assignment and potential confounding variables. However, the potential for residual confounding (factors we did not control for) and confounding by indication always remains in register-based studies. We adjusted for maternal characteristics, pregestational disorders, pregnancy disorders, and covariates associated with an increased risk of preeclampsia or bleeding and receiving prescription for either 150 to 160 mg or 75 mg of aspirin. However, we did not have data to correct for other factors, such as preeclampsia heredity, mean arterial pressure, mean uterine artery pulsatility index on Doppler ultrasound, or biomarkers, all of which are components in the Fetal Medicine Foundation algorithm^19^ that was used in 1 region where the higher dose of aspirin is prescribed. This might explain why we found a slightly increased risk of preeclampsia among nulliparous women using 150 to 160 mg of aspirin compared with those using 75 mg. Furthermore, there is always a risk of random error in studies with multiple outcomes and subanalyses. Among women undergoing cesarean delivery, the risk of postpartum hemorrhage was lower with 150 to 160 mg (2.1%) compared with 75 mg (3.2%) of aspirin. However, bleeding during cesarean delivery was classified as intrapartum rather than postpartum hemorrhage. As a result, most cesarean delivery–related bleeding events were not recorded as postpartum hemorrhage.

Information on aspirin adherence was not available, which may impact the effectiveness and potential complications. Nevertheless, our study reflects the clinical scenario, where aspirin adherence varies and should not differ between groups. Given the inherent nature of observational data, some covariates were incomplete, which may have impacted our findings. However, we are reassured in that the greatest level of missing data for any included covariate was only 6.5% (for information on previous pregnancies for parous women) and no difference in missing patterns by exposure status or outcomes. Lastly, our study was conducted using data from Sweden and thus confirmation of our findings in a population of different ethnicity is warranted and would improve the generalizability of our findings.

## Conclusions

In this large population-based study, we found no difference in either preeclampsia or bleeding complications when comparing 150 to 160 mg and 75 mg of aspirin for the prevention of preeclampsia. Large, randomized trials comparing aspirin doses must include both efficacy and safety as primary outcomes to ensure that any additional benefit of higher doses does not come at the risk of maternal bleeding.

## Notes

Supplement 1. **eAppendix. **Statistical Analysis Plan
eMethods.
**eTable 1.** Source of Information for the Covariates **eTable 2.** Balance Tables **eFigure 1.** Preeclampsia Balance Plots **eFigure 2.** Postpartum Hemorrhage Balance Plots **eTable 3.** Obstetric History Among Parous Women by Aspirin Dosage **eTable 4.** Region by Aspirin Dosage **eTable 5.** Year by Aspirin Dosages **eTable 6.** Main Outcomes by Aspirin Dosage Among Nulliparous Women
eReferences.

Supplement 2.
Data Sharing Statement
