# Supplementary material for: Prophylactic Aspirin Dose and Preeclampsia
Source: JAMA Netw Open. 2025 Feb 3;8(2):e2457828. doi: 10.1001/jamanetworkopen.2024.57828 (PMC11791696; doi:10.1001/jamanetworkopen.2024.57828)
Supplement: Supplement 1. — eAppendix. Statistical Analysis Plan eMethods. eTable 1. Source of Information for the Covariates eTable 2. Balance Tables eFigure 1. Preeclampsia Balance Plots eFigure 2. Postpartum Hemorrhage Balance Plots eTable 3. Obstetric History Among Parous Women by Aspirin Dosage eTable 4. Region by Aspirin Dosage eTable 5. Year by Aspirin Dosages eTable 6. Main Outcomes by Aspirin Dosage Among Nulliparous Women eReferences. [file jamanetwopen-e2457828-s001.pdf]

Supplemental Online Content

Kupka E, Hesselman S, Gunnarsdóttir J, et al. Prophylactic aspirin dose and preeclampsia. *JAMA Netw Open*. 2025;8(1):e2457828. doi:10.1001/jamanetworkopen.2024.57828

eAppendix. Statistical Analysis Plan

eMethods.

eTable 1. Source of Information for the Covariates

eTable 2. Balance Tables

eFigure 1. Preeclampsia Balance Plots

eFigure 2. Postpartum Hemorrhage Balance Plots

eTable 3. Obstetric History Among Parous Women by Aspirin Dosage

eTable 4. Region by Aspirin Dosage

eTable 5. Year by Aspirin Dosage

eTable 6. Main Outcomes by Aspirin Dosage Among Nulliparous Women

eReferences.

This supplemental material has been provided by the authors to give readers additional information about their work.

© 2025 Kupka E et al. *JAMA Network Open*.

eAppendix. Statistical Analysis Plan

Background

There is no international consensus regarding the optimal aspirin dosage for the prevention of preeclampsia. In non-pregnant individuals, doses between 50-100 mg per day of aspirin have been associated with effective inhibition of COX-1, leading to an inhibited platelet generation of thromboxane-A2 and a subsequent antithrombotic effect [1]. But there is evidence that pregnant women have a reduced aspirin concentration in plasma compared to non-pregnant women due to altered pharmacokinetics [2,3]. International guidelines recommend 75 to 150 mg per day and vary between countries and regions [4–6]. In Sweden, women who present with one high-risk factor or several moderate risk factors for preeclampsia are advised to take 75 mg aspirin per day [7]. However, some centers in Sweden have previously or currently prescribe 150/160 mg of aspirin daily to women at high risk of preeclampsia. It is unknown how aspirin prevents preeclampsia, possible mechanisms include COX-1 inhibition[8], by enhancing placental implantation in early pregnancy [9], by lowering peripheral vascular resistance [10], or due to a protective effect of aspirin on endothelial cells and subsequently reduced inflammatory activity [11].

Our team have previously reported an increased risk of intrapartum and postpartum hemorrhage [8], as well as neonatal intracranial haemorrhage with the use of low-dose aspirin in pregnancy [12]. Additionally, a study of 7 infants born to mothers using 100mg of aspirin daily during pregnancy, found that although aspirin concentrations in umbilical cord blood were below the detectable limit, platelet function was impaired [13]. A recent systematic review and meta-analysis concluded that higher doses of aspirin at 150 – 162 mg were more effective than lower doses of 75-80mg. However, this meta-analysis only included 3 studies and had significant methodological limitations, which impede the interpretation of these findings. The authors of this meta-analysis also recognised the need for further high-quality studies to determine the optimal dose of aspirin, where the benefit of preeclampsia prevention is weighed against the potential bleeding risk.

In Sweden, aspirin use in pregnancy is prescribed and data of its use captured within Swedish Prescribed Drug Register. This offers a unique opportunity to investigate aspirin use in pregnancy at a population level and assess rare outcomes. Thus, combing data from large Swedish registers, we will examine the association between the use of 75 mg or 150-160 mg aspirin in pregnancy and the risk of preeclampsia at birth and bleeding complications.

Project overview

This study will be performed in three parts:

1) Descriptive analysis for the overall cohort, pregnant women who use aspirin 75 mg and pregnant women who use aspirin 150-160 mg.
2) We will compare the effect of aspirin 75mg and 150-160 mg on the risk of preeclampsia and bleeding complications. We will use propensity score matching to adjust for differences between the groups.
3) Supplementary analyses of 75 vs 150-160mg of aspirin:
   - sensitivity analysis of only primiparous women
   - bleeding complication by mode of birth.

PICO

P: The study population will consist of all women with aspirin prescription in pregnancy, with at least one pregnancy recorded in the Medical Birth register between 2007-2019. One pregnancy will be randomly selected from each woman.

I/E: Primary exposure will be the use of 150-160 mg aspirin based on prescription data from the Swedish Prescribed Drug register.

C: Women prescribed 75 mg of aspirin, based on prescription data from the Swedish Prescribed Drug register

© 2025 Kupka E et al. *JAMA Network Open*.

O: There will be two primary outcomes: preeclampsia diagnosis (analysis 1) and postpartum hemorrhage (bleeding >1000 ml) (analysis 2). Separate analyses will be run for the two primary outcomes.

Study variables
Demographics and baseline characteristics

Current pregnancy

Age at delivery (years)

Height

Weight

Parity

Country of birth (Nordic, Non-Nordic European, Rest of the world)

Smoking first antenatal visit

Conception method (Spontaneous, Ovulation drugs, In vitro fertilization)

Education (University, Upper secondary school, < 12 years of school attendance)

Pre-gestational disorders (Chronic hypertension, Diabetes [Diabetes type 1, Diabetes type 2], Chronic kidney disease, Systemic lupus erythematosus, Anti-phospholipid syndrome,

Pregnancy-induced disorders)

Gestational diabetes

Placenta previa

Placenta accrete

Placental abruption

Gestational age at delivery

Induction of labor

Mode of delivery (Unassisted vaginal, Instrumental vaginal, Cesarean delivery)

Use of SSRIs

Use of LMWH

Thromboembolic event in previous pregnancies or before partus in current pregnancy

Previous pregnancies

Hypertension

Preeclampsia

Small for gestational age infant

Gestational length at delivery

Placental abruption

© 2025 Kupka E et al. *JAMA Network Open*.

Cesarean delivery

Stillbirth

Exposure variables

Low-dose aspirin during pregnancy (anytime from conception to delivery)

Outcome variables

Primary outcomes

Preeclampsia diagnosis during pregnancy or postpartum

Postpartum bleeding

Secondary outcomes

Preeclampsia analysis

Gestational week at delivery with preeclampsia diagnosis

Preeclampsia with delivery <37 weeks

Preeclampsia with delivery <34 weeks

Preeclampsia with delivery >37 weeks

Preeclampsia with small for gestational age (SGA) infant

Severe preeclampsia

Bleeding analysis

Antepartum hemorrhage

Intrapartum hemorrhage

Postpartum hematoma (superficial/ deep)

Anemia/ blood transfusion

Neonatal intracranial bleeding

Data sources

Medical Birth Register

- Pregnancy variables: conception via *in-vitro* fertilization (including intracytoplasmic sperm injection), preeclampsia, HELLP, placental abruption, placenta previa, placenta accrete, cesarean section, intrapartum bleeding, postpartum bleeding, postpartum hematoma, gestational age of delivery, small for gestational age (SGA) birth and stillbirth. SGA birth will be defined as birthweight below two standard deviations according to Swedish growth charts [14]. Information on conception via *in-vitro* fertilization and stillbirth will

© 2025 Kupka E et al. *JAMA Network Open*.

be retrieved from predefined checkboxes in the Medical Birth Register, self-reported by the women and/or diagnostic codes.

- Maternal demographics: maternal age at delivery, height, body mass index (BMI), smoking (yes/no), country of birth, pregestational disorders (including chronic hypertension, diabetes, chronic kidney disease, systemic lupus erythematosus, antiphospholipid syndrome). Information on pre-gestational disorders will be retrieved from predefined checkboxes in the Medical Birth Register, self-reported by the women and/or diagnostic codes.
- Information about previous births for multiparous women with a previous birth: mean gestational length at delivery (one mean that includes all previous pregnancies), stillbirth, SGA, previous cesarean section, hypertension, preeclampsia, placental abruption in any previous pregnancy pregnancy.
- Neonatal intracranial bleeding (icd code P52, P10)
- Antepartum hemorrhage (icd code O46)
- Intrapartum hemorrhage (icd code O67)
- Postpartum hematoma (superficial hematoma icd code O90.2, T81.0 and deep hematoma icd O717)
- Anemia (icd code 099.0, 0991)
- Postpartum hematoma (superficial hematoma icd code O90.2, T81.0 and deep hematoma icd O717)
- Information about a previous thromboembolic event (icd code I80, I26, O22.3 and 088) and information about a embolic event in the current pregnancy (icd code O88) before partus.

Statistics Sweden

- Information on maternal highest obtained level of education (university, upper secondary school degree, or <12 years of school attendance).

Swedish Prescribed Drug Register

- Information about low-dose aspirin use (ATC code B01AC06), defined as at least one dispensed prescription during pregnancy and included women with a prescription of 75 or 160 mg aspirin from conception.
- Information about Low Molecular Weight Heparin (LMWH) (ACT code B01AB), defined as at least one dispensed prescription during pregnancy and included women with a prescription of LMWH from conception.

National patient register within 42 days after birth

- Postpartum hematoma (superficial hematoma icd code O90.2, T81.0 and deep hematoma icd O717)
- Anemia (icd code 099.0, 0991)
- Blood transfusion during pregnancy DR029
- Information about a previous thromboembolic event (icd code I80, I26, O22.3 and 088) and information about a embolic event in the current pregnancy (icd code O88) before partus.

Descriptive analysis

Flow chart of study participants

Total number in cohort -> number of women with 75 mg aspirin and 150-160 mg aspirin (one pregnancy per women)

© 2025 Kupka E et al. *JAMA Network Open*.

Exclusion criteria : multifetal pregnancies.

Population characteristics to be described for

a. Total cohort
b. 75 mg aspirin
c. 150 – 160 mg aspirin

Statistical analysis

Null hypothesis

Women who use 75 mg of aspirin during pregnancy and women who use 150-160 mg of aspirin during pregnancy will have the same risk of preeclampsia. Women who use 75 mg of aspirin during pregnancy and women who use 150-160 mg of aspirin during pregnancy will have the same risk of bleeding complications at delivery.

Anticipated sample size:

All women with aspirin prescription

		Frequency	Percent	Valid Percent	Cumulative Percent
Valid	1,00	9721	82,7	82,7	82,7
	2,00	2037	17,3	17,3	100,0
	Total	11758	100,0	100,0	

1= aspirin 75 mg, 2= aspirin 150-160 mg

Preeclampsia diagnosis among women with aspirin prescription

		Frequency	Percent	Valid Percent	Cumulative Percent
Valid	0	10592	90,1	90,1	90,1
	1	1166	9,9	9,9	100,0
	Total	11758	100,0	100,0	

0= no preeclampsia, 1= preeclampsia

Postpartum hemorrhage (PPH) among women with aspirin prescription

© 2025 Kupka E et al. *JAMA Network Open*.

		Frequency	Percent	Valid Percent	Cumulative Percent
Valid	0	11007	93,6	93,6	93,6
	1	751	6,4	6,4	100,0
	Total	11758	100,0	100,0	

0= no PPH, 1= PPH

With a prevalence of 9,9% of preeclampsia in the 75 mg aspirin group, the power for the preeclampsia outcome is 80,5 using 20% difference (decrease) and 99% using 30% difference (decrease).

With a prevalence of 6,4% of PPH in the 75 mg aspirin group, the power for the PPH outcome is 56% using 20% difference (increase) and 86% using 30% difference (increase).

Inclusion criteria: all women with at least one pregnancy recorded in the Medical Birth register between 2007-2019 who were prescribed aspirin 75 mg or 150-160 mg in the first trimester.

Exclusion criteria: multiple pregnancies.

The effect of aspirin 75 mg compared to 150-160 mg of aspirin will be estimated. This will be presented as a risk ratio and risk difference with corresponding 95% confidence intervals.

A propensity score model will be created for each outcome with the final included covariates determined by the authorship team and informed by directed acyclic graphs. The background characteristics from the two groups will guide the creation of the direct acyclic graphs. One propensity score will be created for the primary outcome preeclampsia one propensity score will be created for the primary outcome postpartum hemorrhage. We will perform an inverses probability weighted regression adjustment using the propensity score derived above.

A. Regression modelling will then be used to obtain effect estimates.
B. Both adjusted and unadjusted risk ratios and risk difference will be reported including 95% confidence intervals for all models.
C. We will calculate the absolute risk of the main outcomes to make the results more understandable and communicable

Analysis:

Potential estimators are (i) selection model, using IPW; (ii) outcome model, using regression adjustment; (iii) doubly robust IPWRA models. Under correct specification, all the estimators should produce similar results. We will focus on IPWRA; selection model will be included in the IPW and outcome in RA model specification. We will combine both regression adjustment and augmented IPW in a doubly robust regression model. We will also perform RA, IPW and multivariate LR separately.

Model for exposure assignment using inverse probability weighted propensity scores derived from the logistic exposure model. Adequacy of balance achieved by PS weighting will be assessed using: (i) standardized mean difference for model covariates and variance estimates between exposure groups and (ii) propensity scores overlap. If unbalanced we will explore measures such as: addition of interaction terms; different exposure models; use of alternate weighting structures (overlap or stabilized); and lastly propensity score-based trimming with the aim of achieving overlap if the propensity score distributions.

DAG: association between aspirin dosage and preeclampsia

© 2025 Kupka E et al. *JAMA Network Open*.

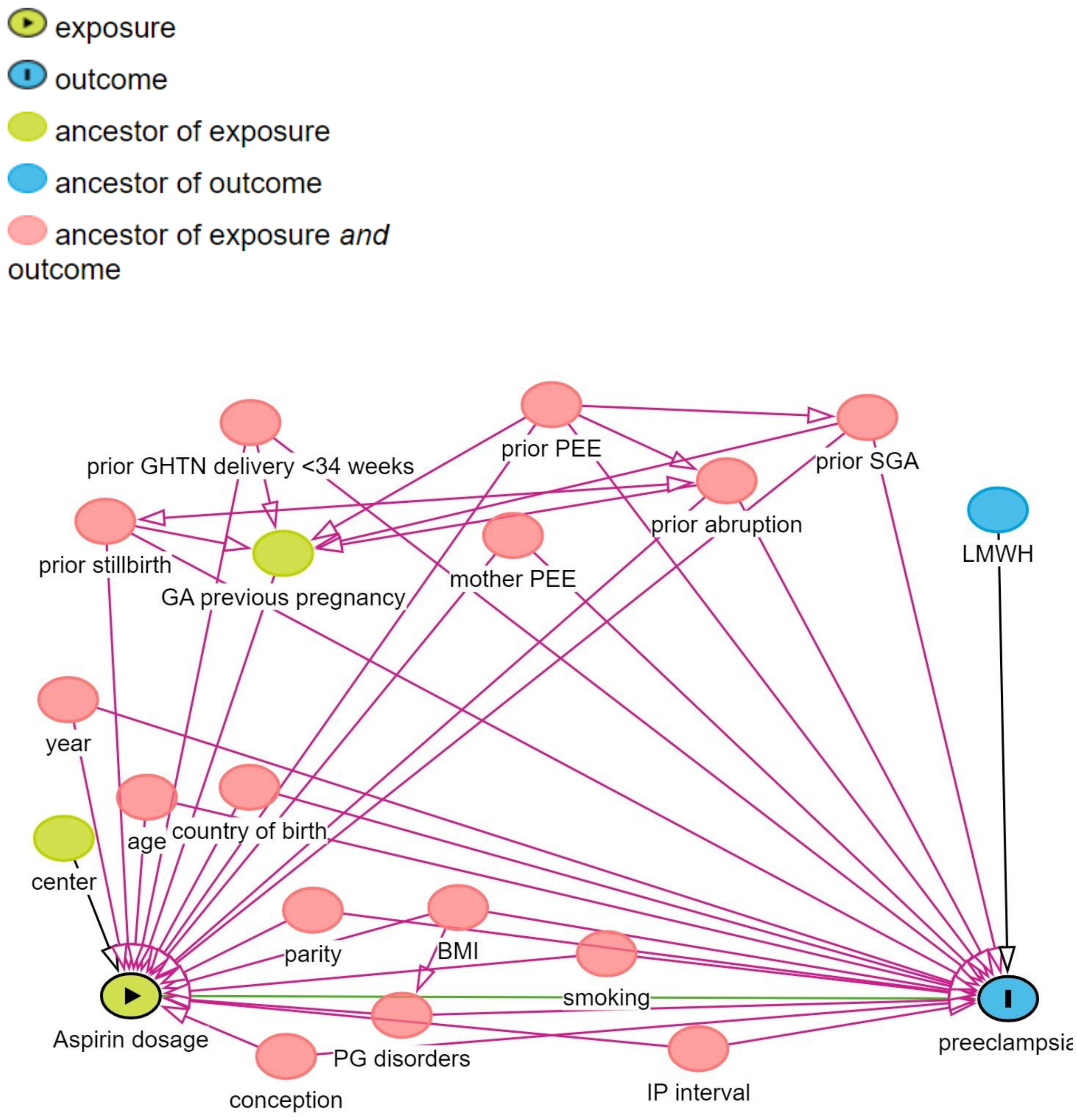

GHTN, gestational hypertension; GA, gestational age; PG, pregestational; BMI, body mass index; PEE, preeclampsia; IP, interpregnancy interval; SGA, small for gestational age; LMWH, low molecule weight heparin.

DAG: association between aspirin dosage and postpartum bleeding, antepartum bleeding and intrapartum bleeding

© 2025 Kupka E et al. *JAMA Network Open*.

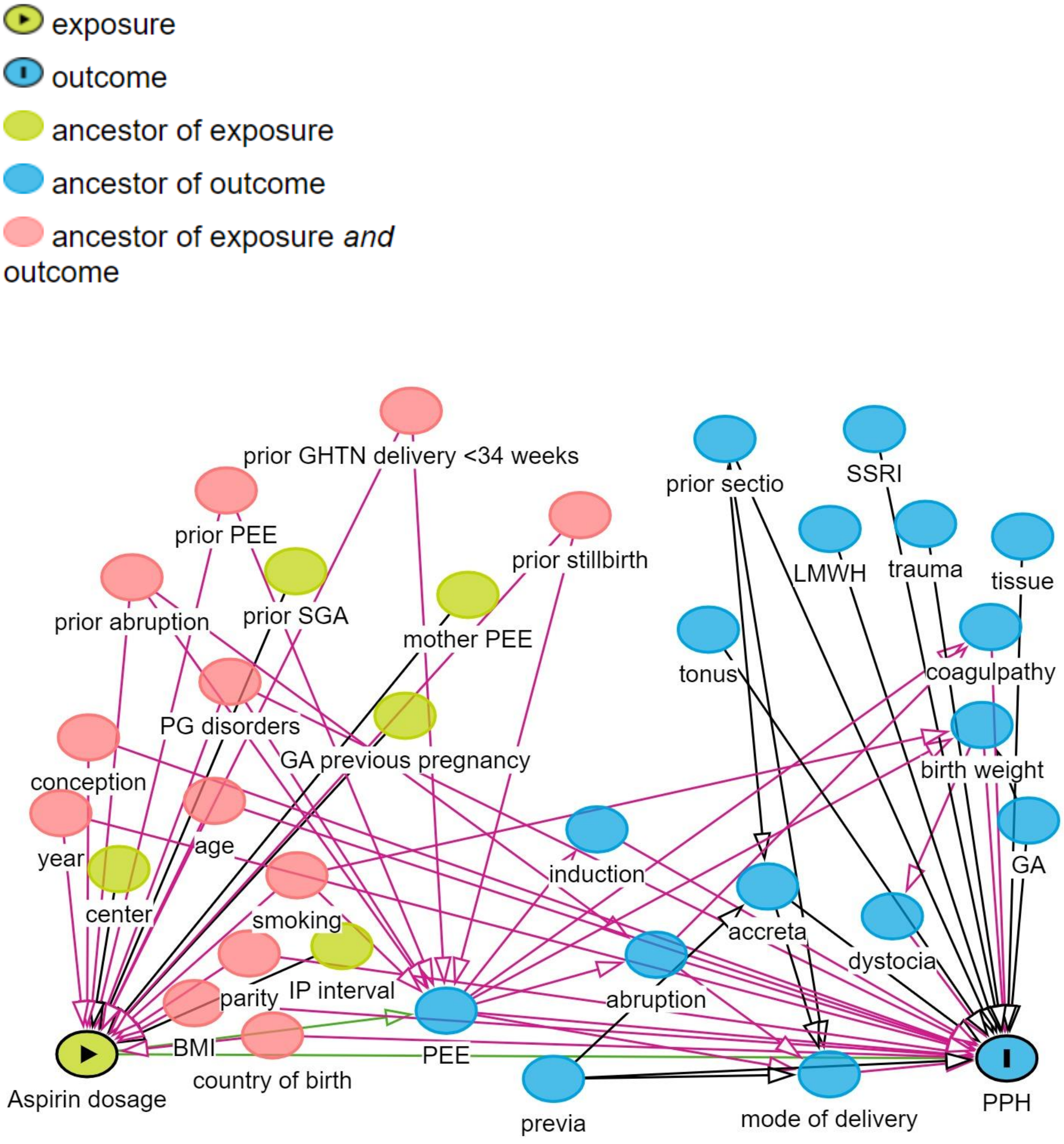

GHTN, gestational hypertension; GA, gestational age; PG, pregestational; BMI, body mass index; PEE, preeclampsia; IP, interpregnancy interval; SGA, small for gestational age; LMWH, low molecule weight heparin; SSRI, Selective serotonin reuptake inhibitor.

DAG: association between aspirin dosage and postpartum hematoma

© 2025 Kupka E et al. *JAMA Network Open*.

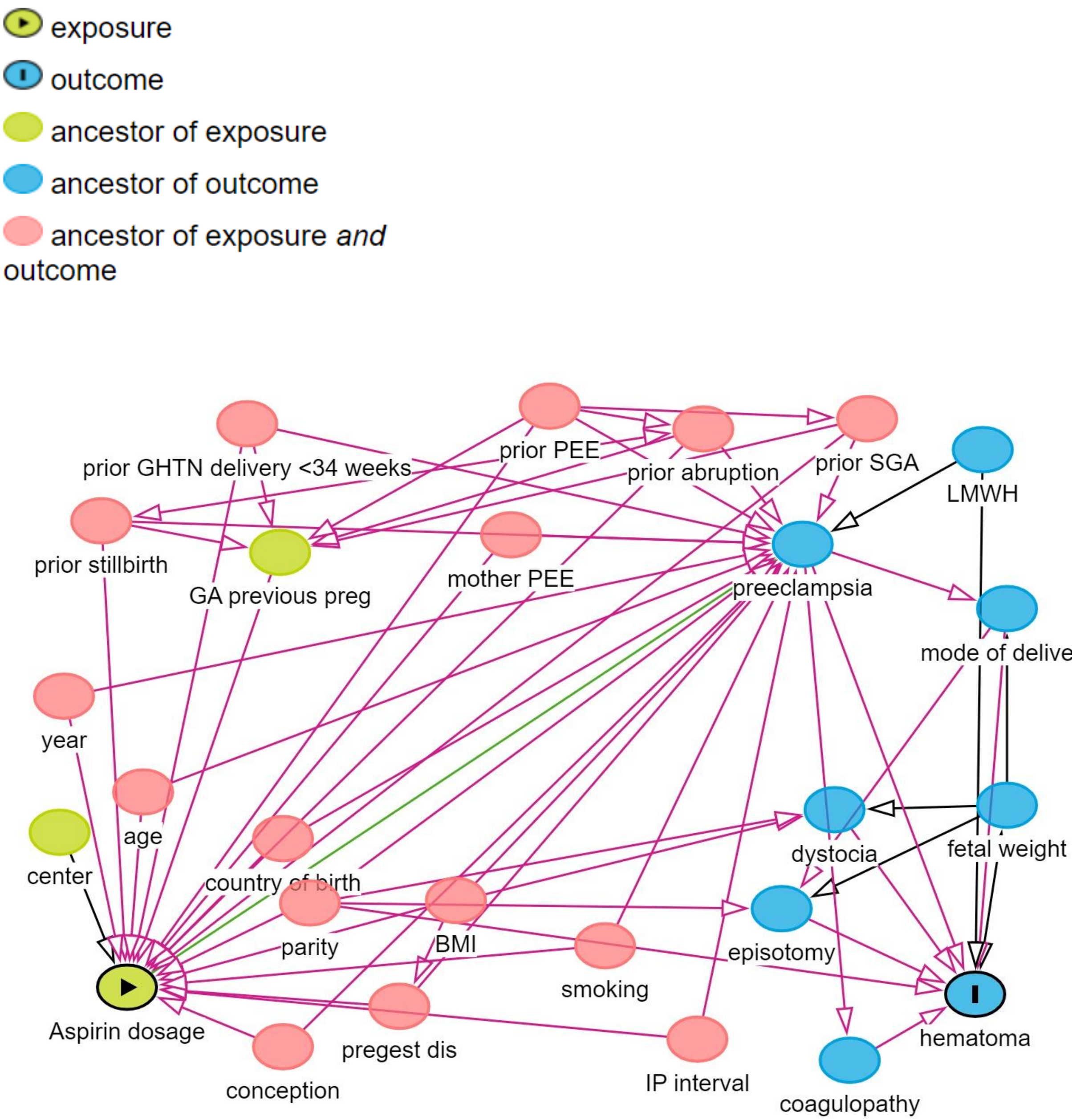

GHTN, gestational hypertension; GA, gestational age; BMI, body mass index; PEE, preeclampsia; IP, interpregnancy interval; SGA, small for gestational age; LMWH, low molecule weight heparin.

DAG: association between aspirin dosage and neonatal intracranial hemorrhage

© 2025 Kupka E et al. *JAMA Network Open*.

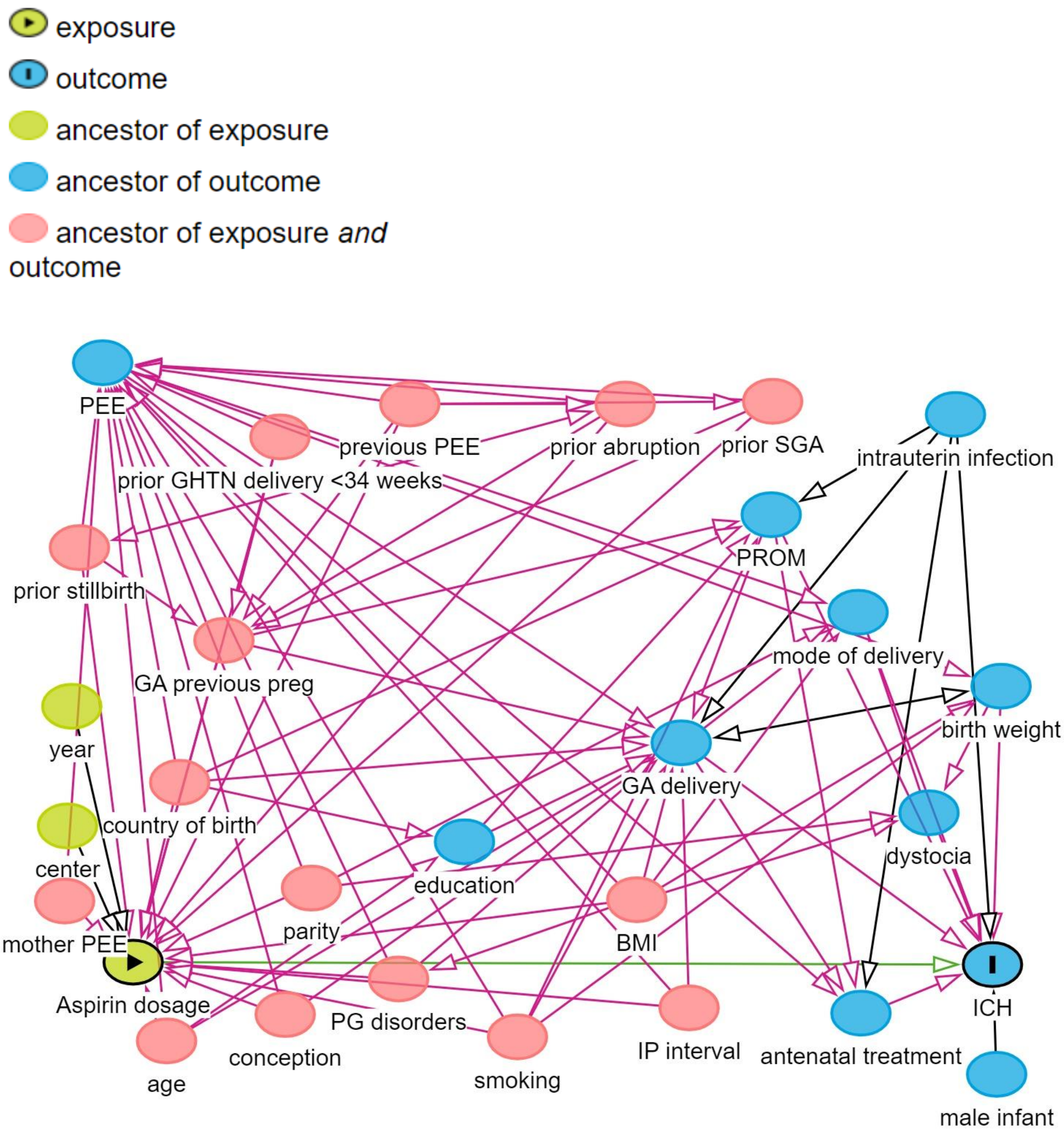

ICH, intracranial hemorrhage; GHTN, gestational hypertension; GA, gestational age; BMI, body mass index; PEE, preeclampsia; IP, interpregnancy interval; SGA, small for gestational age; PROM, premature rupture of membranes.

DAG: association between aspirin dosage and anaemia

© 2025 Kupka E et al. *JAMA Network Open*.

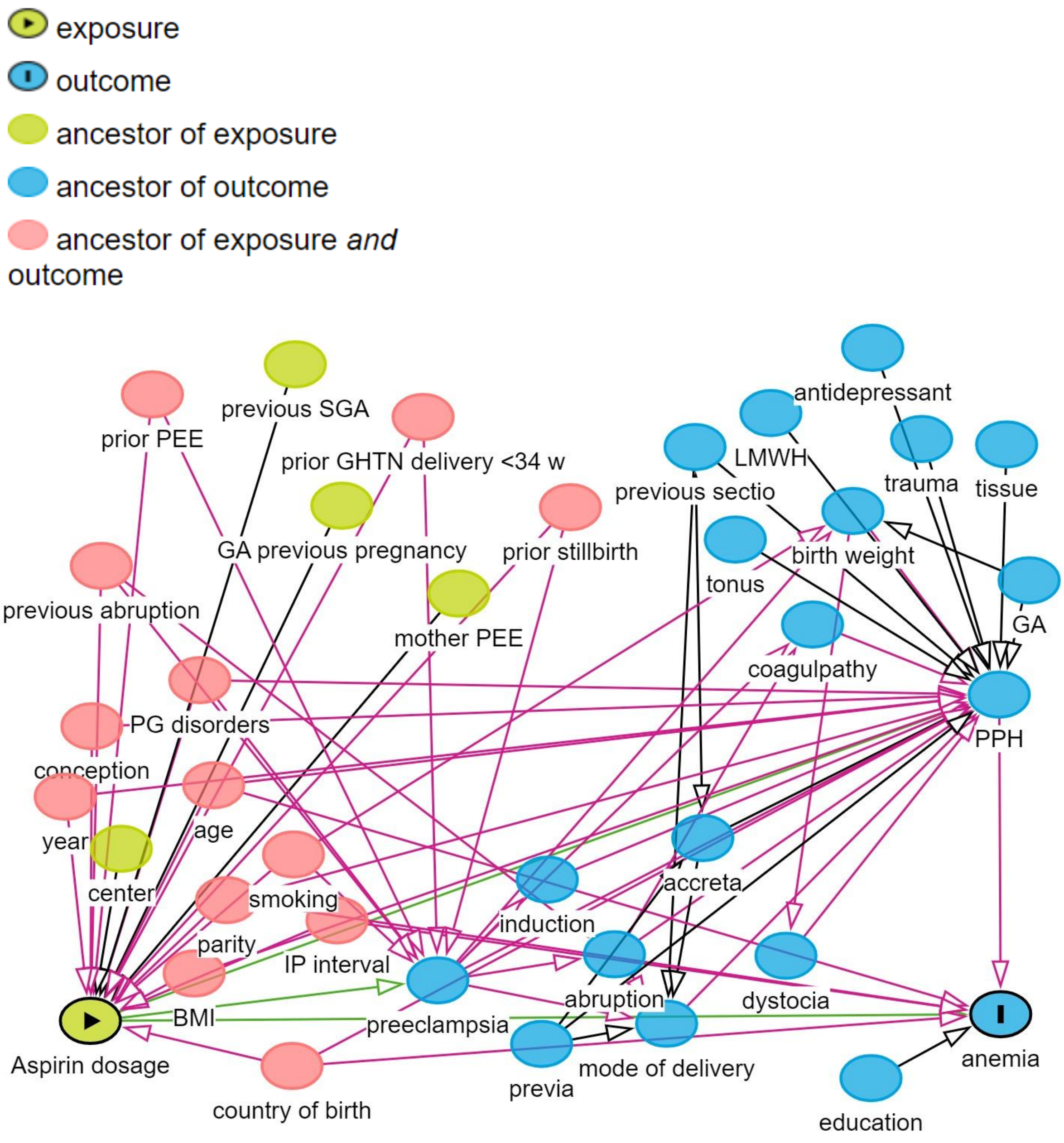

GHTN, gestational hypertension; GA, gestational age; PG, pregestational; BMI, body mass index; PEE, preeclampsia; IP, interpregnancy interval; SGA, small for gestational age; PPH, postpartum hemorrhage; LMWH, low molecule weight heparin.

Table 2: primary and secondary analyses

Variable	Aspirin 75 mg (n=xx)	Aspirin 150-160 mg (n=yy)	Relative risk (95% confidence interval)		Absolute risk (95% confidence interval)	
			Crude	Adjusted*	Crude	Adjusted*
**Primary outcomes**						

© 2025 Kupka E et al. *JAMA Network Open*.

Preeclampsia diagnosis	n (%)	n (%)	Xx (xx.x–xx.x), p=0.xxxx	xx.x (xx.x–xx.x), p=0.xxxx	xx.x (xx.x–xx.x), p=0.xxxx	xx.x (xx.x–xx.x), p=0.xxxx
Postpartum hemorrhage	n (%)	n (%)	xx.x (xx.x–xx.x), p=0.xxxx	xx.x (xx.x–xx.x), p=0.xxxx	xx.x (xx.x–xx.x), p=0.xxxx	xx.x (xx.x–xx.x), p=0.xxxx
**Secondary outcomes**						
Gestational week at delivery with preeclampsia diagnosis	xx.x (xx.x–xx.x),	xx.x (xx.x–xx.x),	x.xx (x.xx–x.xx), p=0.xxxx	x.xx (x.xx–x.xx), p=0.xxxx		
Preeclampsia with delivery <37 weeks	n (%)	n (%)	Xx (xx.x–xx.x), p=0.xxxx	Xx (xx.x–xx.x), p=0.xxxx		
Preeclampsia with delivery <34 weeks	n (%)	n (%)	Xx (xx.x–xx.x), p=0.xxxx	Xx (xx.x–xx.x), p=0.xxxx		
Preeclampsia with delivery >37 weeks	n (%)	n (%)	Xx (xx.x–xx.x), p=0.xxxx	Xx (xx.x–xx.x), p=0.xxxx		
Preeclampsia with small for gestational age infant	n (%)	n (%)	x.xx (x.xx–x.xx), p=0.xxxx	x.xx (x.xx–x.xx), p=0.xxxx		
Severe preeclampsia						
Antepartum hemorrhage	n (%)	n (%)	x.xx (x.xx–x.xx), p=0.xxxx	x.xx (x.xx–x.xx), p=0.xxxx		
Intrapartum hemorrhage	n (%)	n (%)	x.xx (x.xx–x.xx), p=0.xxxx	x.xx (x.xx–x.xx), p=0.xxxx		
Postpartum hematoma	n (%)	n (%)	x.xx (x.xx–x.xx), p=0.xxxx	x.xx (x.xx–x.xx), p=0.xxxx		
Neonatal intracranial bleeding	n (%)	n (%)	x.xx (x.xx–x.xx), p=0.xxxx	x.xx (x.xx–x.xx), p=0.xxxx		
Anemia/ transfusion						

For primary outcomes, data are presented as number and percent, 95% confidence interval for proportion, and relative risk and absolute risk with 95% CI.

For secondary outcomes, data are presented as median and interquartile range, number and percent, 95% confidence interval for proportion, and relative risk with 95% CI.

*Adjusted for … For postpartum hemorrhage, any maternal bleeding event and neonatal intracranial bleeding we adjusted for…

Supplementary Table 2: sensitivity analysis of only primiparous women

© 2025 Kupka E et al. *JAMA Network Open*.

Variable	Aspirin 75 mg (n=xx)	Aspirin 150-160 mg (n=yy)	Relative risk (95% confidence interval)	
			Crude	Adjusted*
Preeclampsia diagnosis	n (%)	n (%)	Xx (xx.x–xx.x), p=0.xxxx	xx.x (xx.x–xx.x), p=0.xxxx
Postpartum hemorrhage	n (%)	n (%)	xx.x (xx.x–xx.x), p=0.xxxx	xx.x (xx.x–xx.x), p=0.xxxx

Data are presented as number and percent, 95% confidence interval for proportion, and relative risk with 95% CI.

*Adjusted for…

Supplementary Table 3: sensitivity analysis of the cohort between 2017-2020

Variable	Aspirin 75 mg (n=xx)	Aspirin 150-160 mg (n=yy)	Relative risk (95% confidence interval)	
			Crude	Adjusted*
Preeclampsia diagnosis	n (%)	n (%)	Xx (xx.x–xx.x), p=0.xxxx	xx.x (xx.x–xx.x), p=0.xxxx
Postpartum hemorrhage	n (%)	n (%)	xx.x (xx.x–xx.x), p=0.xxxx	xx.x (xx.x–xx.x), p=0.xxxx

Data are presented as number and percent, 95% confidence interval for proportion, and relative risk with 95% CI.

*Adjusted for…

Supplementary Table 4: bleeding complication by mode of birth.

Outcome	Vaginal deliveries				Cesarean deliveries			
	Aspirin 75 mg (n=xx)	Aspirin 150-160 mg (n=yy)			Aspirin 75 mg (n=xx)	Aspirin 150-160 mg (n=yy)		
	N (%)	N (%)	RR (95% CI) Crude	Adjusted*	N (%)	N (%)	RR (95% CI) Crude	Adjusted*
Antepartum hemorrhage								
Intrapartum hemorrhage								
Postpartum hemorrhage								

© 2025 Kupka E et al. *JAMA Network Open*.

Postpartum hematoma								
Neonatal intracranial bleeding								
Anemia								

Data are presented as number and percent, 95% confidence interval for proportion, and relative risk with 95% CI.

*Adjusted for…

Variables included in statistical analyses

**Variable**	**Variable name in data file**	**Comment**
**Background characteristics current pregnancy**		
Age at delivery (years)	MALDER	16-55
≥35	MALDERkat	0= <34 years, 1= ≥35
Body mass index (kg/m^2) [a]	BMI	15.6-63.3
Body mass index ≥30	BMI_cat	0-3 <30, 4-6 ≥30
Height	MLANGD	139-192
Weight	MVIKT	36-179
Parity	PARITETkat	0=primiparous<br>1= second or third birth<br>2= fourth birth or more<br>≥ 4
Country of birth	MFODLANDkat	1=Nordic<br>2=Non-Nordic European<br>3=Rest of the world<br>Missing
Smoking first antenatal visit	Smoking2	Smoking at first antenatal visit<br>0= no<br>1= yes
Conception method	IVF2	IVF (IVF and or/ICSI)<br>0= no ivf<br>1= ivf
	ostim	Ovulation stimulation<br>0=no stim<br>1= ovulation stimulation
	IVF3	0= spontaneus conception<br>1= not spontaneus (IVF and or ICSI and or ovulation simulation)
Education	Utbildning	1=University<br>2=Upper secondary school<br>3=< 12 years of school attendance<br>0= information missing
Chronic hypertension[a]	hypertension	0= no hypertension<br>1= pregestational hypertension

© 2025 Kupka E et al. *JAMA Network Open*.

Diabetes[a]	diabetskorr	0= no diabetes<br>1= pregestational diabetes
Diabetes type 1	dia_diab1_m	0= no diabetes type 1<br>1= diabetes type 1
Diabetes type 2	dia_diab2_m	0= no diabetes type 2<br>1= diabetes type 2
Pregnancy diabetes	Preg_diab	0= no pregnancy diabetes<br>1= pregnancy diabetes
Chronic kidney disease	njursjukdom	0= no<br>1 = chronic kidney disease
Systemic lupus erythematosus[a]	SLE2	0=n0<br>1= yes
Anti-phospholipid syndrome[a]	dia_antifossyndrom_m	0=no<br>1= yes
Placental abruption	dia_ablatio_m	0=no<br>1=yes
Placenta previa	dia_previa_m	0=no<br>1=yes
Gestational age at delivery	GRVBS	22-43
Induction of labor	Dia_induction_m	0=no<br>1=yes
Mode of delivery	f_slut	1=Unassisted vaginal<br>2=Instrumental vaginal<br>3=Cesarean delivery
Use of antidepressants	atc_antidepressiva_	0=no<br>1=yes
Use of LMWH during pregnancy or a thromboembolism diagnosis	Lmwh_tromb	0=no<br>1=yes
Region	region	1=Region Stockholm<br>2=Region Uppsala<br>3=Region Sörmland<br>4=Region Östergötland<br>5=Region Jönköping<br>6=Region Kronoberg<br>7=Region Kalmar<br>8=Region Gotland<br>9=Region Blekinge<br>10=Region Skåne<br>11=Region Halland<br>12=Region Västra Götaland<br>13=Region Värmland<br>14=Region Örebro<br>15=Region Västmanland<br>16=Region Dalarna<br>17=Region Gävleborg<br>18=Region Västernorrland<br>19=Region Jämtland Härjedalen<br>20=Region Västerbotten<br>21=Region Norrbotten
**Previous pregnancies**		
Preeclampsia	X_preeklampsi_m	0=no<br>1=yes

© 2025 Kupka E et al. *JAMA Network Open*.

Small for gestational age infant	x_MSGA_m	0=no<br>1=yes
Placental abruption	X_ablatio_m	0=no<br>1=yes
Cesarean delivery	TSECTIO	0=no<br>1=yes
Stillbirth	X_IUFD	0= no stillbirth<br>1= stillbirth

Variable	Variable name in data file	Comment
**Exposure variables**		
Low-dose aspirin during pregnancy (from conception)	ASA_ug_all	0=75 mg<br>1=150-160 mg
**Primary outcomes**		
Preeclampsia diagnosis during pregnancy or postpartum	dia_preeclampsia2_m	0= no preeclampsia<br>1= preeclampsia
Postpartum bleeding	dia_pph_m	0= no postpartum bleeding<br>1= postpartum bleeding
**Secondary outcomes**		
Gestational week at delivery with preeclampsia diagnosis		
Preeclampsia with delivery <37 weeks	PEE_below37	0= no<br>1= yes
Preeclampsia with delivery <34 weeks	PEE_below34	0=no<br>1=yes
Preeclampsia with delivery >=37 weeks	PEE_above37	0=no<br>1=yes
Preeclampsia with small for gestational age (SGA) infant	PEE_SGA	0=no<br>1=yes
Antepartum hemorrhage	Dia_ant_hemorrage_m	0=no<br>1=yes
Intrapartum hemorrhage	Dia_intrapart_hemorrage_m	0=no<br>1=yes
Postpartum hematoma	Dia_djupt_hematom_mfr_m	0=no<br>1=yes
Neonatal intracranial bleeding	Dia_neo_ch_b	0=no<br>1=yes
Anemia	Dia_anemi_pm_during	0=no<br>1=yes
**Confounders**		
IP interval (months)	IP	0-322
Age at delivery (years)	MALDER	16-55
Body mass index (kg/m^2) [a]	BMI	15.6-63.3
Parity	PARITET_F	1-13
Country of birth	MFODLANDkat	1=Nordic<br>2=Non-Nordic European<br>3=Rest of the world

© 2025 Kupka E et al. *JAMA Network Open*.

		Missing
Smoking first antenatal visit	Smoking2	Smoking at first antenatal visit<br>0= no<br>1= yes
Conception method	IVF2	IVF (IVF and or/ICSI)<br>0= no ivf<br>1= ivf
Chronic hypertension[a]	hypertension	0= no hypertension<br>1= pregestational hypertension
Diabetes type 1	dia_diab1_m	0= no diabetes type 1<br>1= diabetes type 1
Diabetes type 2	dia_diab2_m	0= no diabetes type 2<br>1= diabetes type 2
Chronic kidney disease	njursjukdom	0= no<br>1 = chronic kidney disease
Systemic lupus erythematosus[a]	SLE2	0=n0<br>1= yes
Anti-phospholipid syndrome[a]	dia_antifossyndrom_m	0=no<br>1= yes
Year	AR	2007-2020
Prev Preeclampsia	X_preeklampsi_m	0=no<br>1=yes
Prev Small for gestational age infant	x_MSGA_m nu x_MSGA	0=no<br>1=yes
Prev Placental abruption	X_ablatio_m	0=no<br>1=yes
Prev Cesarean delivery	TSECTIO	0=no<br>1=yes
Prev Stillbirth	X_IUFD	0= no stillbirth<br>1= stillbirth
Prev gestational hypertension with delivery below 34 weeks gestation	x_gesthyp_34w	0=no<br>1=yes

© 2025 Kupka E et al. *JAMA Network Open*.

eMethods

Covariates

Information on maternal demographics was obtained from the Medical Birth Register and included year, maternal age at delivery, body mass index (BMI), parity, country of birth (Nordic, non-Nordic European, and rest of the world), smoking (yes/no) and the geographical region of the maternity unit where the women gave birth. The highest education level (university, upper secondary school degree, or less than 12 years of school attendance) was retrieved from Statistics Sweden. Information on pregnancy variables was also obtained from the Medical Birth Register and included conception via in-vitro fertilization (including intracytoplasmic sperm injection), pregestational disorders (chronic hypertension, diabetes, chronic kidney disease, systemic lupus erythematosus, antiphospholipid syndrome), gestational diabetes, placental abruption, placenta previa, placenta accreta, labour dystocia, intrapartum infection, premature rupture of membranes, induction of labour, mode of delivery (spontaneous vaginal, instrumental vaginal, caesarean delivery), gestational age at delivery, birth weight, small for gestational age infant, and stillbirth. Small for gestational age infant was defined as birth weight more than two standard deviations below the mean birthweight for gestational age (i.e. the 2.5th percentile), according to Swedish growth charts [14].

For parous women, we retrieved information on chronic hypertension, diabetes, preeclampsia, placental abruption, caesarean delivery, gestational age at delivery, and small for gestational age-infant and stillbirths in previous pregnancies recorded in the Medical Birth Register. Information about previous pregnancies was missing in 708 of 10868 parous women (6.5%).

Information on thromboembolic events before and during the index pregnancy was obtained from the Medical Birth Register and National Patient Register. Data on antidepressant and Low Molecular Weight Heparin (LMWH) use, defined as at least one dispensed prescription from three months before conception until delivery, was obtained from the Swedish Prescribed Drug Register. Since some centers in Sweden provide LMWH without a prescription, the variable reflecting LMWH use during pregnancy was defined as an LMWH prescription, an/or a thromboembolic event before or during the index pregnancy.

© 2025 Kupka E et al. *JAMA Network Open*.

eTable 1. Source of information for the covariates

Register	Variables	Information
**Medical Birth Register**	Cardiovascular disease	ICD code I63, I21, I252, O994
	**Covariates**	
	Year	Antenatal care record
	Age	Antenatal care record
	Body mass index	Antenatal care record
	Parity	Antenatal care record
	Country of birth	Antenatal care record (based on the pre-defined categories: Africa, Asia, North America, Europe, Oceania, Other Nordic Countries, Other Unspecified countries, the former Soviet Union, South America, Sweden)
	Smoking first antenatal visit	Antenatal care record, pre-defined checkbox, self-reported by the mother
	Region	Birth record
	Conception method	Antenatal care record, pre-defined checkbox, self-reported by the mother
	Chronic hypertension	Antenatal care record, pre-defined checkbox, self-reported by the mother
	Diabetes type 1	ICD code O240
	Diabetes type 2	ICD code O241
	Chronic kidney disease	Antenatal care record, pre-defined checkbox, self-reported by the mother
	Systemic lupus erythematosus	Antenatal care record, pre-defined checkbox, self-reported by the mother
	Anti-phospholipid syndrome	ICD code D686A
	Gestational diabetes	ICD code O244, O249
	Placental abruption	ICD code O45, O46
	Placenta previa	ICD code O44
	Placenta accreta	ICD code O432
	Labor dystocia	ICD code O62, O63
	Intrapartum infection	ICD code O752, O753, O411
	Premature rupture of membranes	ICD code O42
	Induction of labour	ICD code O61
	Mode of delivery	Birth record, pre-defined checkbox

© 2025 Kupka E et al. *JAMA Network Open*.

	Gestational age at delivery	Birth record
	Neonatal birth weight	Birth record
	Small for gestational age infant	Birth record
	Stillbirth	Birth record
	Previous caesarean section	Birth record, pre-defined checkbox
	Previous stillbirth	Antenatal care record
	**Outcomes**	
	Preeclampsia	ICD code O14, O15, O11
	Postpartum haemorrhage	ICD code O72
	Antepartum haemorrhage	ICD code O46
	Intrapartum haemorrhage	ICD code O67
	Postpartum hematoma	ICD code O717, O902
	Neonatal intracranial bleeding	ICD code P52, P10
**Medical Birth Register and National Patient Register**	**Covariates**	
	Thromboembolism in the index pregnancy	ICD code O23, O88
	Any thromboembolic event before the index pregnancy	ICD code O23, O88, I80
	**Outcome**	
	Anaemia	ICD code O990, O991
**The Prescribed Drug Register**	**Covariates**	
	Antidepressant prescription	ATC code N06A
	LMWH prescription	ATC code B01AB
	**Exposure**	
	Low-dose aspirin prescription	ATC code B01AC06
**Statistic Sweden**	Education	-

© 2025 Kupka E et al. *JAMA Network Open*.

eTable 2A-D. Balance tables

2A. Treatment model

Treatment model		
**Variable**	**Standardized differences**	
	**Raw**	**Weighted**
Pre-gestational disorder	0.0653035	0.0242896
Prior small for gestational age infant	0.3094652	-0.0300578
Prior abruption	0.2945106	-0.0300578
Prior preeclampsia	0.3490621	-0.0094176
Prior gestational hypertension with birth <34 weeks'	0.3223604	-0.0359421
Prior intrauterine foetal death	0.2773431	0.0148698
Gestational age of prior birth	-0.295265	0.0168499
Maternal age	-0.2412081	-0.0096068
Body mass index	0.2146758	.018391
Parity	-0.2200304	0.0188255
Smoking	0.0513979	-0.0049109
In vitro fertilization	-0.1311781	-0.0182045
Maternal country of birth	-0.1163355	-0.028454
Interpregnancy interval > 10 years	0.3242634	-0.0349352
Year of birth		
2018	0.1817372	-0.0170114
2019	0.442311	-0.0155628
2020	-0.2008562	0.017106
Region	0.4980161	0.0613815

2B. Treatment model: women with a vaginal birth

Treatment model		
**Variable**	**Standardized differences**	
	**Raw**	**Weighted**
Pre-gestational disorder	0.0609456	0.0161704

© 2025 Kupka E et al. *JAMA Network Open*.

Prior pregnancy complications	0.2820391	0.0005386
Prior small for gestational age infant	0.2951534	-0.0216333
Gestational age of prior birth	-0.2812098	0.0186898
Maternal age	-0.1748878	0.0054767
Body mass index	0.2219788	0.019508
Parity	-0.2054873	0.0174059
Smoking	0.0572991	-0.0106319
In vitro fertilization	-0.1229603	-0.0018086
Maternal country of birth	-0.114011	-0.017
Interpregnancy interval > 10 years	0.3114767	-0.0292289
Year of birth		
2018	0.1922586	-0.0125143
2019	0.4387027	-0.0149057
2020	-0.2011586	0.0191855
Region	0.4480777	0.0483417

2C. Treatment model: women with a caesarean section birth

**Treatment model**		
**Variable**	**Standardized differences**	
	**Raw**	**Weighted**
Pre-gestational disorder	0.1022377	0.0087555
Prior pregnancy complications	0.3353706	-0.0015221
Prior small for gestational age infant	0.3501027	-0.0399105
Gestational age of prior birth	-0.3493824	0.0426243
Maternal age	-0.3247355	-0.0268511
Body mass index	0.2628605	0.0098723
Parity	-0.2639245	0.0446077
Smoking	0.0413286	0.0413936
In vitro fertilization	-0.1323534	-0.0420073
Maternal country of birth	-0.0942458	-0.0468665
Interpregnancy interval > 10 years	0.364569	-0.0614883
Year of birth		
2018	0.144211	-0.0434915

© 2025 Kupka E et al. *JAMA Network Open*.

2019	0.4388123	-0.012839
2020	-0.1584625	0.0236204
Region	0.144211	-0.0434915

2D. Treatment model: nulliparous women

**Among nulliparous**		
**Variable**	**Standardized differences**	
	**Raw**	**Weighted**
Pregestational disorder	0.0317235	0.0466532
Maternal age	-0.3241424	0.0268681
Body mass index	0.4336767	0.0139549
Smoking	0.0900629	-0.0083095
In vitro fertilization	-0.3245058	0.0047073
Maternal country of birth	-0.296564	-0.0306005
**Year**		
2018	0.2064881	-0.0020443
2019	0.4366873	0.0151977
Region	0.5242191	0.0407358

© 2025 Kupka E et al. *JAMA Network Open*.

eFigure 1A-C. Preeclampsia Balance plots

1A. Preeclampsia Balance plot: Body Mass Index

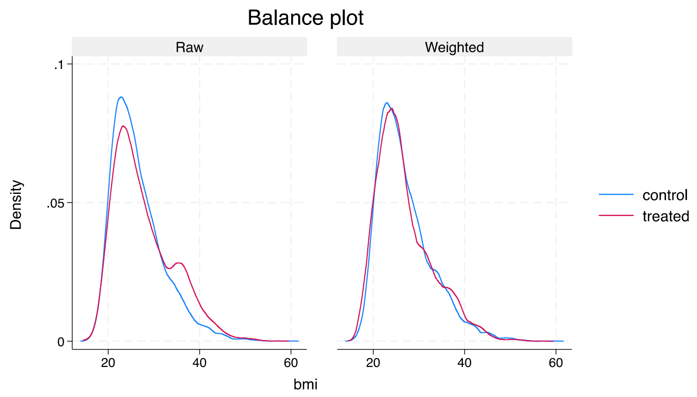

1B. Preeclampsia Balance plot: maternal age

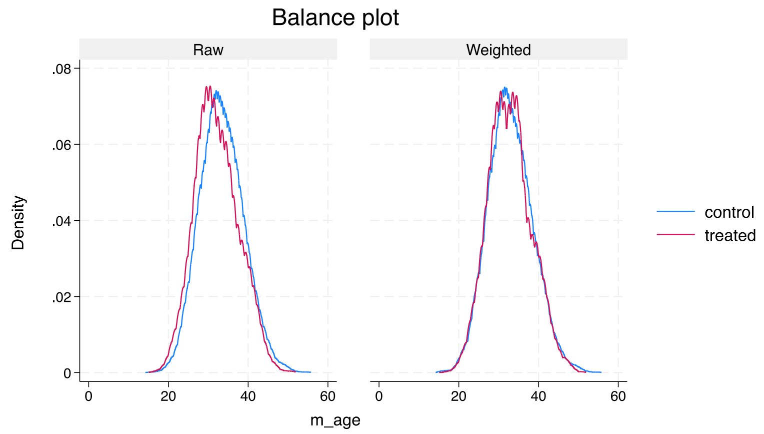

1C. Preeclampsia Balance plot: parity

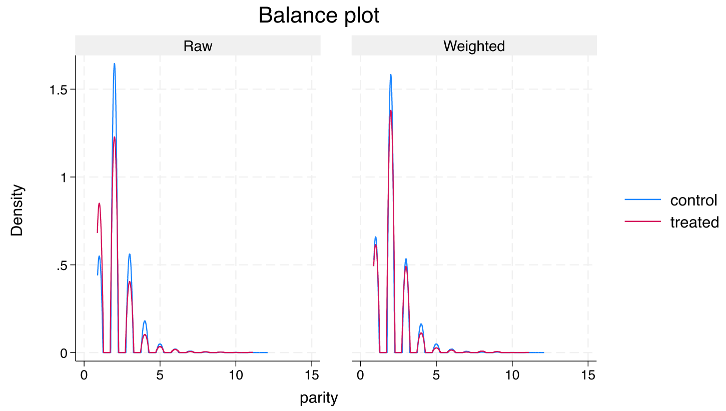

© 2025 Kupka E et al. *JAMA Network Open*.

eFigure 2A-C: Postpartum haemorrhage Balance plots

2A. Postpartum haemorrhage Balance plot: Body Mass index

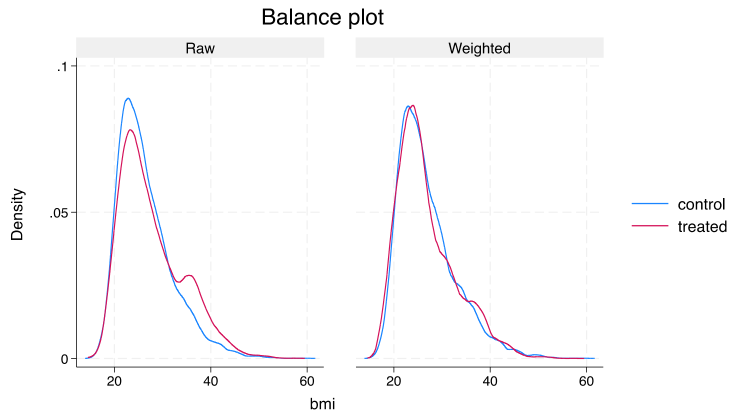

2B. Postpartum haemorrhage Balance plot: maternal age

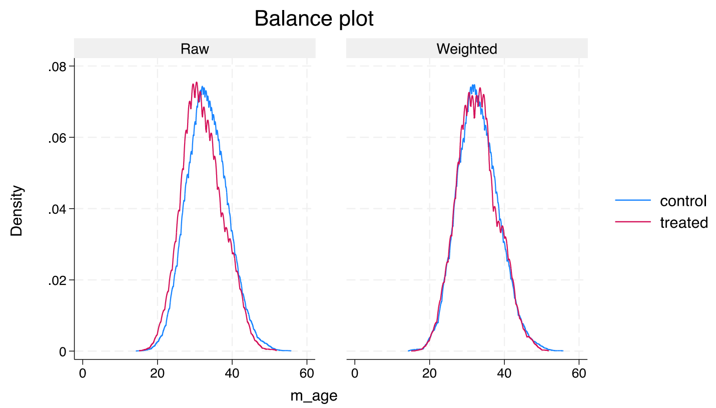

2C. Postpartum haemorrhage Balance plot: parity

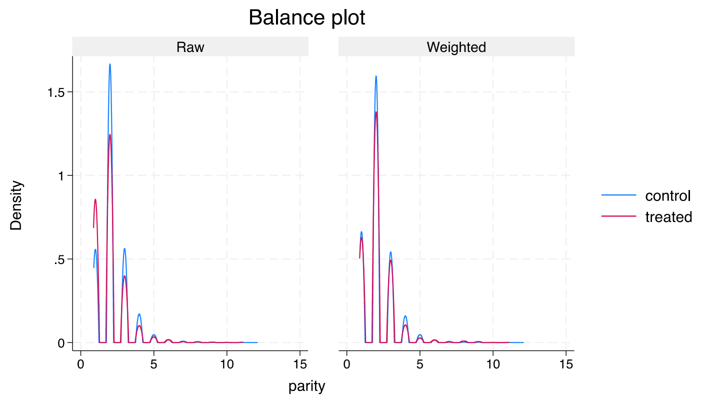

© 2025 Kupka E et al. *JAMA Network Open*.

eTable 3. Obstetric History Among Parous Women by Aspirin Dosage

		Aspirin use	
**Obstetric history***	**Total parous women (n=10825)**<br><br>**N (%)**	**150-160 mg (N=3267)**<br><br>**N (%)**	**75 mg (n= 7558)**<br><br>**N (%)**
Previous preeclampsia, n (%)	3871 (35.8)	1327 (40.6)	2544 (33.7)
Previous placental abruption, n (%)	420 (3.9)	76 (2.3)	344 (4.6)
Previous small for gestational age infant (≤2.5th percentile), n (%)	2557 (23.6)	815 (24.9)	1742 (23.0)
Previous caesarean sectio, n (%)	4351 (41.9)	1091 (35.0)	3260 (44.8)
Previous stillbirth, n (%)	823 (7.6)	169 (5.2)	654 (8.7)

Data are presented as n (%) or mean ± SD

* The diagnosis in any previous pregnancy

© 2025 Kupka E et al. *JAMA Network Open*.

eTable 4. Region by Aspirin Dosage

		Aspirin use	
**Region**	**Total births (n= 13,828)**<br><br>**N (%)**	**150-160 mg (n= 4,687)**<br><br>**N (%)**	**75 mg (n= 9,141)**<br><br>**N (%)**
Region Stockholm	3702 (26.8)	53 (1.1)	3649 (39.9)
Region Uppsala	324 (2.3)	11 (0.2)	313 (3.4)
Region Sörmland	262 (1.9)	52 (1.1)	210 (2.3)
Region Östergötland	1088 (7.9)	622 (13.3)	466 (5.1)
Region Jönköping	477 (3.5)	329 (7.0)	148 (1.6)
Region Kronoberg	140 (1.0)	6 (0.1)	134 (1.5)
Region Kalmar	456 (3.3)	240 (5.1)	216 (2.4)
Region Gotland	62 (0.4)	2 (0.0)	60 (0.7)
Region Blekinge	152 (1.1)	22 (0.5)	130 (1.4)
Region Skåne	1371 (9.9)	429 (9.2)	942 (10.3)
Region Halland	488 (3.5)	196 (4.2)	292 (3.2)
Region Västra Götaland	3427 (24.8)	2658 (56.7)	769 (8.4)
Region Värmland	267 (1.9)	44 (0.9)	223 (2.4)
Region Örebro	280 (2.0)	3 (0.1)	277 (3.0)
Region Västmanland	147 (1.1)	0 (0.0)	147 (1.6)
Region Dalarna	229 (1.7)	4 (0.1)	225 (2.5)
Region Gävleborg	201 (1.5)	3 (0.1)	198 (2.2)
Region Västernorrland	210 (1.5)	2 (0.0)	208 (2.3)
Region Jämtland Härjedalen	42 (0.3)	0 (0.0)	42 (0.5)
Region Västerbotten	287 (2.1)	7 (0.1)	280 (3.1)
Region Norrbotten	211 (1.5)	3 (0.1)	208 (2.3)
Missing	5 (0.0)	1 (0.0)	4 (0.0)

Data are presented as n (%) or mean ± SD.

© 2025 Kupka E et al. *JAMA Network Open*.

eTable 5. Year by Aspirin Dosage

		Aspirin use	
Year	Total births (n= 13,828) N (%)	150-160 mg n= 4,687 N (%)	75 mg n= 9,141 N (%)
2017	2367 (17.1)	177 (3.8)	2190 (24.0)
2018	3443 (24.9)	1400 (29.9)	2043 (22.3)
2019	4449 (32.2)	2133 (45.5)	2316 (25.3)
2020	3569 (25.8)	977 (20.8)	2592 (28.4)

Data are presented as n (%) or mean ± SD.

© 2025 Kupka E et al. *JAMA Network Open*.

eTable 6. Main Outcomes by Aspirin Dosage Among Nulliparous Women

	Aspirin 150-160 mg (n=1420)<br><br>N (%)	Aspirin 75 mg (n=1583) reference<br>N (%)	Relative risk (95% confidence interval)	
			Crude	Adjusted
**Preeclampsia**	156 (11.0)	159 (10.0)	1.09 (0.89-1.35)	1.40 (1.05-1.87)
**Postpartum haemorrhage**	123 (8.7)	148 (9.4)	0.93 (0.74-1.16)	1.18 (0.83-1.68)

Frequencies (n) and percent (%).

n=3,003 included in the crude model. n=2,753 included in the adjusted model for preeclampsia and n= 2,831 included in the adjusted model for postpartum haemorrhage. A doubly robust inverse probability weighted regression adjustment model was used for the adjusted analysis with aspirin 75 mg as reference.

© 2025 Kupka E et al. *JAMA Network Open*.

eReferences

1. Gurbel PA, Bliden KP, DiChiara J, et al. Evaluation of dose-related effects of aspirin on platelet function: results from the Aspirin-Induced Platelet Effect (ASPECT) study. *Circulation*. 2007;115(25):3156-3164. doi:10.1161/CIRCULATIONAHA.106.675587

2. Shanmugalingam R, Wang X, Münch G, et al. A pharmacokinetic assessment of optimal dosing, preparation, and chronotherapy of aspirin in pregnancy. *Am J Obstet Gynecol*. 2019;221(3):255.e1-255.e9. doi:10.1016/j.ajog.2019.04.027

3. Aspirin non-responsiveness in pregnant women at high-risk of pre-eclampsia - ClinicalKey. Accessed August 13, 2021. https://www.clinicalkey.com/#!/content/playContent/1-s2.0-S0301211517306048?returnurl=null&referrer=null

4. US Preventive Services Task Force. Aspirin Use to Prevent Preeclampsia and Related Morbidity and Mortality: US Preventive Services Task Force Recommendation Statement. *JAMA*. 2021;326(12):1186-1191. doi:10.1001/jama.2021.14781

5. Overview | Hypertension in pregnancy: diagnosis and management | Guidance | NICE. Accessed May 2, 2021. https://www.nice.org.uk/guidance/ng133

6. Poon LC, Shennan A, Hyett JA, et al. The International Federation of Gynecology and Obstetrics (FIGO) initiative on pre-eclampsia: A pragmatic guide for first-trimester screening and prevention. *Int J Gynaecol Obstet Off Organ Int Fed Gynaecol Obstet*. 2019;145 Suppl 1:1-33. doi:10.1002/ijgo.12802

7. *Riktlinjer För Hypertonisjukdomar under Graviditet, SFOG 2019-10-23. Reviderad 210121*. https://www.sfog.se/media/337263/hypertonisjukdomar-under-graviditet-sfog-2019-10-23-reviderad-210121.pdf

8. Duley L, Meher S, Hunter KE, Seidler AL, Askie LM. Antiplatelet agents for preventing pre-eclampsia and its complications. *Cochrane Database Syst Rev*. 2019;2019(10). doi:10.1002/14651858.CD004659.pub3

9. Atallah A, Lecarpentier E, Goffinet F, Doret-Dion M, Gaucherand P, Tsatsaris V. Aspirin for Prevention of Preeclampsia. *Drugs*. 2017;77(17):1819-1831. doi:10.1007/s40265-017-0823-0

10. Helgadóttir H, Tropea T, Gizurarson S, Mandalà M. Endothelium-Derived Hyperpolarizing Factor (EDHF) Mediates Acetylsalicylic Acid (Aspirin) Vasodilation of Pregnant Rat Mesenteric Arteries. *Int J Mol Sci*. 2021;22(18):10162. doi:10.3390/ijms221810162

11. Dzeshka MS, Shantsila A, Lip GYH. Effects of Aspirin on Endothelial Function and Hypertension. *Curr Hypertens Rep*. 2016;18(11):83. doi:10.1007/s11906-016-0688-8

12. Hastie R, Tong S, Wikström AK, Sandström A, Hesselman S, Bergman L. Aspirin use during pregnancy and the risk of bleeding complications: a Swedish population-based cohort study. *Am J Obstet Gynecol*. 2021;224(1):95.e1-95.e12. doi:10.1016/j.ajog.2020.07.023

13. Leonhardt A, Bernert S, Watzer B, Schmitz-Ziegler G, Seyberth HW. Low-dose aspirin in pregnancy: maternal and neonatal aspirin concentrations and neonatal prostanoid formation. *Pediatrics*. 2003;111(1):e77-81. doi:10.1542/peds.111.1.e77

14. Marsál K, Persson PH, Larsen T, Lilja H, Selbing A, Sultan B. Intrauterine growth curves based on ultrasonically estimated foetal weights. *Acta Paediatr Oslo Nor 1992*. 1996;85(7):843-848. doi:10.1111/j.1651-2227.1996.tb14164.x

© 2025 Kupka E et al. *JAMA Network Open*.
