# Supplementary material for: Prophylactic Aspirin Dose and Preeclampsia
Source: JAMA Netw Open. 2025 Feb 3;8(2):e2457828. doi: 10.1001/jamanetworkopen.2024.57828 (PMC11791696; doi:10.1001/jamanetworkopen.2024.57828)
Supplement: Supplement 2. — Data Sharing Statement [file jamanetwopen-e2457828-s002.pdf]

Data Sharing Statement

Kupka. Prophylactic Aspirin Dose and Preeclampsia. *JAMA Netw Open*. Published February 03, 2025. doi:10.1001/jamanetworkopen.2024.57828

Data
Data available: No
